# Saccharified Uranyl Ions: Self‐Assembly of UO_2_^2+^ into Trinuclear Anionic Complexes by the Coordination of Glucosamine‐Derived Schiff Bases

**DOI:** 10.1002/chem.202100546

**Published:** 2021-05-21

**Authors:** Gerrit Schaper, Marco Wenzel, Felix Hennersdorf, Leonard F. Lindoy, Jan J. Weigand

**Affiliations:** ^1^ Faculty of Chemistry and Food Chemistry Technische Universität Dresden 01062 Dresden Germany; ^2^ School of Chemistry F11 University of Sydney NSW 2006 Sydney Australia

**Keywords:** biologically inspired ligands, glucosamine-derived Schiff bases, polynuclear coordination complexes, uranyl, X-ray diffraction

## Abstract

The reaction of UO_2_(OAc)_2_ ⋅ 2H_2_O with the biologically inspired ligand 2‐salicylidene glucosamine (H_2_ **L^1^**) results in the formation of the anionic trinuclear uranyl complex [(UO_2_)_3_(*μ_3_*‐O)(**L^1^**)_3_]^2−^ (**1** ^2−^), which was isolated in good yield as its Cs‐salt, [Cs]_2_ **1**. Recrystallization of [Cs]_2_ **1** in the presence of 18‐crown‐6 led to formation of a neutral ion pair of type [M(18‐crown‐6)]_2_ **1**, which was also obtained for the alkali metal ions Rb^+^ and K^+^ (M=Cs, Rb, K). The related ligand, 2‐(2‐hydroxy‐1‐naphthylidene) glucosamine (H_2_ **L^2^**) in a similar procedure with Cs^+^ gave the corresponding complex [Cs(18‐crown‐6)]_2_[(UO_2_)_3_(*μ_3_*‐O)(**L^2^**)_3_ ([Cs(18‐crown‐6)]_2_ **2**). From X‐ray investigations, the [(UO_2_)_3_O(**L^n^**)_3_]^2−^ anion (n=1, 2) in each complex is a discrete trinuclear uranyl species that coordinates to the alkali metal ion via three uranyl oxygen atoms. The coordination behavior of H_2_ **L^1^** and H_2_ **L^2^** towards UO_2_^2+^ was investigated by NMR, UV/Vis spectroscopy and mass spectrometry, revealing the in situ formation of the **1** ^2−^ and **2** ^2−^dianions in solution.

## Graphical abstract

**Investigation of the coordination behavior** of salicylidene glucosamine (H_2_ **L^1^**) towards uranyl ions (UO_2_^2+^) revealed the exclusive formation of a trinuclear anionic uranyl complex [(UO_2_)_3_(*μ_3_*‐O)(**L^1^**)_3_]^2−^ (**1** ^2−^), which was isolated by precipitation with the alkali metals ions Cs^+^, Rb^+^, and K^+^. Recrystallization of the Cs salt in the presence of 18‐crown‐6 in DMF led to the formation of the solvated ion pair [Cs(18‐crown‐6)]_2_ **1**, in which coordination of the Cs^+^ ion by three uranyl oxygen atoms was observe.

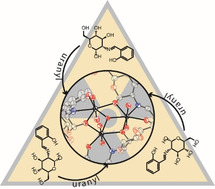

## Introduction

As the most prevalent and thermodynamically stable form of uranium, the coordination chemistry of the uranyl(VI) dication (UO_2_^2+^) continues to be of widespread interest, especially with regards to the use of uranium for civil and military applications and because of its related environmental impact.^[1]^ UO_2_^2+^ is linear, with additional ligands normally coordinating in the equatorial plane and most commonly resulting in a bipyramidal coordination geometry.^[2]^ In both solution and the solid state the tendency of UO_2_^2+^ ions to form polynuclear complexes is well established, very often forming dinuclear and trinuclear species^[3]^ with, in solution, multiple uranyl species frequently occurring together in equilibrium.^[4]^ In aqueous solution at pH=3–5 mononuclear [UO_2_]^2+^, dinuclear [(UO_2_)_2_(OH)_2_]^2+^, and trinuclear [(UO_2_)_3_(OH)_5_]^+^ as well as [(UO_2_)_3_(OH)_4_]^2+^ ions have been reported to coexist.[^4b^, ^4c^, ^4d^, ^4e^, ^4f^, ^4k^, ^4l^, ^4m^, ^4n^] However DFT calculations for [(UO_2_)_3_(OH)_5_]^+^ have shown [(UO_2_)_3_(*μ_3_*‐O)(OH)_3_]^+^, which is indistinguishable by potentiometric titration from the former, to be the most stable geometry for trinuclear uranyl species in solution.^[3a]^ This structural motif was first observed in the solid state as [(UO_2_)_3_(*μ_3_*‐O)(OH)_3_(H_2_O)_6_]NO_3_ ⋅ 4H_2_O by Åberg.^[5]^

Since then, only a handful of such oxo‐bridged uranyl complexes incorporating additional ligands have been isolated. These include triketonates,^[6]^ citrate^[7]^ as well as various salicylidene Schiff bases,^[8]^ with their complexes being synthesized from uranyl hydrate salts employing either aqueous solution or organic solvents.

**Scheme 1:**
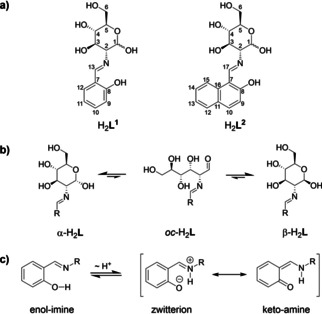
a) Glucosamine Schiff bases used in this study: 2‐salicylidene glucosamine (H_2_ **L^1^**) and 2‐(2‐hydroxy‐1‐naphthylidene) glucosamine (H_2_ **L^2^**) including numbering of carbon atoms; b) anomeric equilibrium of the carbohydrate moiety present for both ligands; c) tautomeric equilibria of the aromatic imine moiety, exemplified for H_2_ **L^1^**.

In recent years the metal coordination chemistry of carbohydrates has attracted much attention, since the latter provide a pool of naturally occurring, enantiomerically pure compounds.^[9]^ Early work of Stephen Angyal *et al*. showed the complexation of several different metal cations, including lanthanides, by a variety of different carbohydrates in solution.^[10]^ Moreover, the adsorption of heavy metal ions by polysaccharide materials is also well established in the literature.^[11]^ Specifically, chitin and chitosan have been previously investigated as possible adsorbents for uranyl ions.^[12]^ The coordination of Pd(II) and Pt(II) by the 2‐glucosamine monomer has been investigated[^9b^, ^13^] while, in particular, glucosamine derived Schiff bases have been reported to act as chelating ligands towards Co(II), Cu(II), Zn(II) as well as the oxo cations V(V)O, Tc(V)O and Mo(VI)O_2_.^[14]^ Such ligands generally form strong complexes that bind in a *κ*_3_‐fashion to the respective metal centers. The ease of modification of the amine‐ and carbonyl‐containing reagents used to form the Schiff base ligands enables the ready tuning of the latter's steric and electronic properties as well as, in turn, those of the resulting metal complexes. While the coordination chemistry of the uranyl ion towards a wide range of Schiff base ligands has now been well documented, and especially towards derivatives of salen,^[15]^ little attention has been given to carbohydrate Schiff base derivatives. In 2001 Sah *et al*.^[16]^ published the crystal structure of a dinuclear uranyl complex containing Schiff base ligands derived from 4,6‐O‐ethylidene‐*β*‐d‐glucopyranosyl‐1‐amine. This to our knowledge is the only crystal structure reported for a carbohydrate‐derived Schiff base uranyl complex. In this report we present the synthesis of new trinuclear, anionic uranyl complexes incorporating the previously reported glucosamine‐derived Schiff bases H_2_ **L^1^** and H_2_ **L^2^** (Scheme 1a).^[17]^

## Results and Discussion

H_2_ **L^1^** and H_2_ **L^2^** were prepared *via* a known procedures,[^17^, ^18^] and are present as their *α*‐ and *β*‐anomers in solution, with both these forms existing in equilibrium *via* the corresponding open chain (*oc*) carbohydrate moiety (Scheme 1b).^[19]^ Further, it has also been established that *o*‐hydroxyaryl Schiff bases exist in solution in tautomeric equilibrium between their enol‐imine form, a zwitterionic form, and the keto‐amine form *via* a proton shift mechanism (Scheme 1c).^[20]^ The ^1^H‐NMR spectrum of H_2_ **L^1^** indicates that the tautomeric equilibrium favors the enol‐imine form (*δ*(α‐**H**C=N)=8.48 ppm, singlet, Figure S1),^[18]^ unlike H_2_ **L^2^**, for which the keto‐amine form is favored (*δ*(α‐**H**C−NH)=8.92 ppm, doublet, ^3^ *J*_17H,NH_=11.5 Hz, Figure S3).^[18]^ The single crystal X‐ray structure of *α*‐H_2_ **L^2^**, published by Mitra *et al*. confirms the expected connectivity.^[21]^ The observed differences between the *o*‐hydroxy benzyl and *o*‐hydroxy naphthyl Schiff bases are in agreement with previously examined systems.[^20c^, ^20g^, ^20h^]

**Figure 1:**
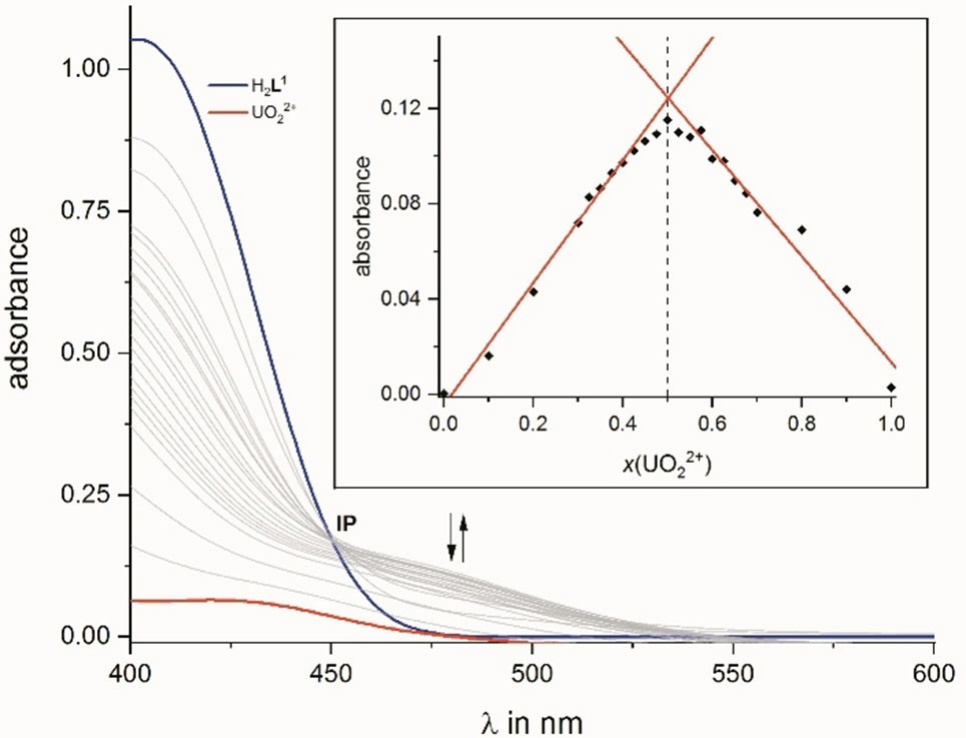
UV/Vis Job Plot for the interaction of H_2_ **L^1^** and uranyl acetate (UO_2_(OAc)_2_ ⋅ 2H_2_O) in MeOH; the absorbance at 480 nm is plotted as a function of the UO_2_^2+^ molar ratio.

We recorded a UV/Vis‐Job plot of H_2_ **L^1^** with UO_2_^2+^ in methanol indicating that an increase of the molar ratio (x) of uranyl ions (x(UO_2_^2+^)) in solution towards x=0.5 led to the emergence of a shoulder at ∼480 nm which we attributed to complex formation. The shoulder is significantly red shifted with respect to both the free ligand and uranyl acetate absorption in methanol (Figure 1). Upon further increase towards x=1.0, the intensity of the shoulder decreases until it disappears. Plotting the absorbance against the molar ratio clearly shows maximum absorbance at x=0.5, corresponding to a metal to ligand (M : L) ratio of M : L=1 : 1 for the complex formed (Figure 1). Furthermore, the UV/Vis plot exhibits an isosbestic point (IP) at λ=450 nm indicating the formation of a single complex species in solution. Similar behavior was observed for the UV/Vis‐Job plot of H_2_ **L^2^** with UO_2_^2+^ in methanol (Figure S15).^[18]^

**Figure 2:**
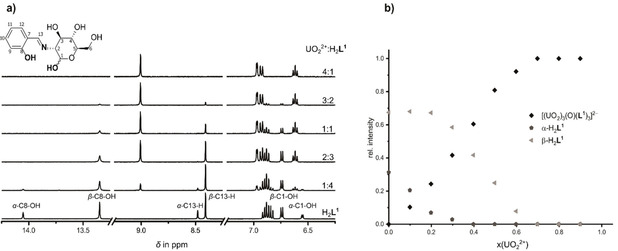
a) Excerpts of the ^1^H‐NMR spectra obtained during the investigation of complex formation between H_2_ **L^1^** and UO_2_(OAc)_2_ ⋅ 2H_2_O in DMSO‐*d_6_*, employing the method of continuous variation; b) Relative intensities of the C13‐H resonance for the species shown with respect to the increasing molar ratio (x(UO_2_^2+^)).

To investigate complex formation further, an NMR investigation was undertaken, once again utilizing Job's method but this time employing DMSO‐*d_6_* as solvent. Excerpts of the ^1^H NMR spectra for selected M : L ratios are shown in Figure 2. The spectrum of the free H_2_ **L^1^** shows two sets of resonances which are assigned to the two anomers, *α*‐H_2_ **L^1^** and *β*‐H_2_ **L^1^**.

As was mentioned above, the dominant pyranose forms of the ligand are in anomeric equilibrium with one another via the open chain form (*oc*‐H_2_ **L^1^**), with the latter constituting less than one percent in equilibrium (Scheme 1b). The observed anomeric ratio of *α*/*β*=0.45 is in agreement with the results from previous NMR investigations.^[20h]^ Upon increasing the M : L ratio with respect to UO_2_(OAc)_2_ ⋅ 2H_2_O, the resonances due to free H_2_ **L^1^** disappear along with the simultaneous emergence of a new set of resonances that correspond to formation of a single metal complex (Figure 2a). On complexation, the resonances for the two hydroxy protons C1−OH and C8−OH of both anomers disappear, in keeping with ligand deprotonation occurring at these positions and subsequent metal complexation via the resulting dianionic di‐alcoholate ligand. In addition, a significant downfield shift for each C13‐H (Schiff base) proton was observed: from *δ*=8.5 ppm for α‐H_2_ **L^1^** and *δ*=8.3 ppm for *β*‐H_2_ **L^1^** to *δ*=9.0 ppm for the resulting metal complex. These observations are also in keeping with the coordination of the imine nitrogen. Comparison of the relative intensities of the C13−H resonances reveal significant differences in reactivity between the two anomers. While the relative abundance of the uranyl complex increases linearly, the abundance of the respective anomers decreases differently. Initially, at low uranyl molar ratios the relative amount of the *β*‐H_2_ **L^1^** is essentially constant, while the molar ratio of *α*‐H_2_ **L^1^** decreases significantly. Only at higher uranyl concentrations, when no more *α*‐H_2_ **L^1^** remains, is a decrease in the relative amount of the *β*‐anomer observed and this continues until finally only the metal complex is present. This clearly indicates that the uranyl ion favors complexation with *α*‐H_2_ **L^1^** over *β*‐H_2_ **L^1^**, with the latter transforming into the former as complexation proceeds via shifts in the equilibrium shown in Scheme 1b. Again, for the Job‐plot of H_2_ **L^2^** and UO_2_^2+^ similar behavior to that for H_2_ **L^1^** was observed (Figure S16, S17).^[18]^

**Scheme 2:**
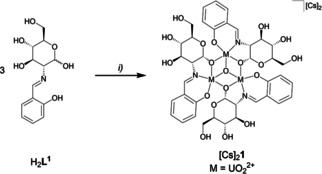
Synthesis of [Cs]_2_ **1**: *i*) 1. H_2_ **L^1^** (4.5 eq), UO_2_(OAc)_2_ ⋅ 2H_2_O (3.0 eq.), MeOH, rt, 2 h; 2. Cs_2_CO_3_ (2.0 eq.), rt, 22 h, yield: 88 %.

The uranyl complex formed could be isolated in good yield by the addition of an alkali metal ion. Adding two equivalents of cesium carbonate to a methanol solution of H_2_ **L^1^** (4.5 eq.) and UO_2_(OAc)_2_ ⋅ 2H_2_O (3.0 eq.) leads to the precipitation of a yellow solid, which was attributed to formation of the trinuclear uranyl complex [Cs]_2_[(UO_2_)_3_(*μ_3_*‐O)(**L^1^**)_3_] ([Cs]_2_ **1**, Scheme 2).

**Figure 3:**
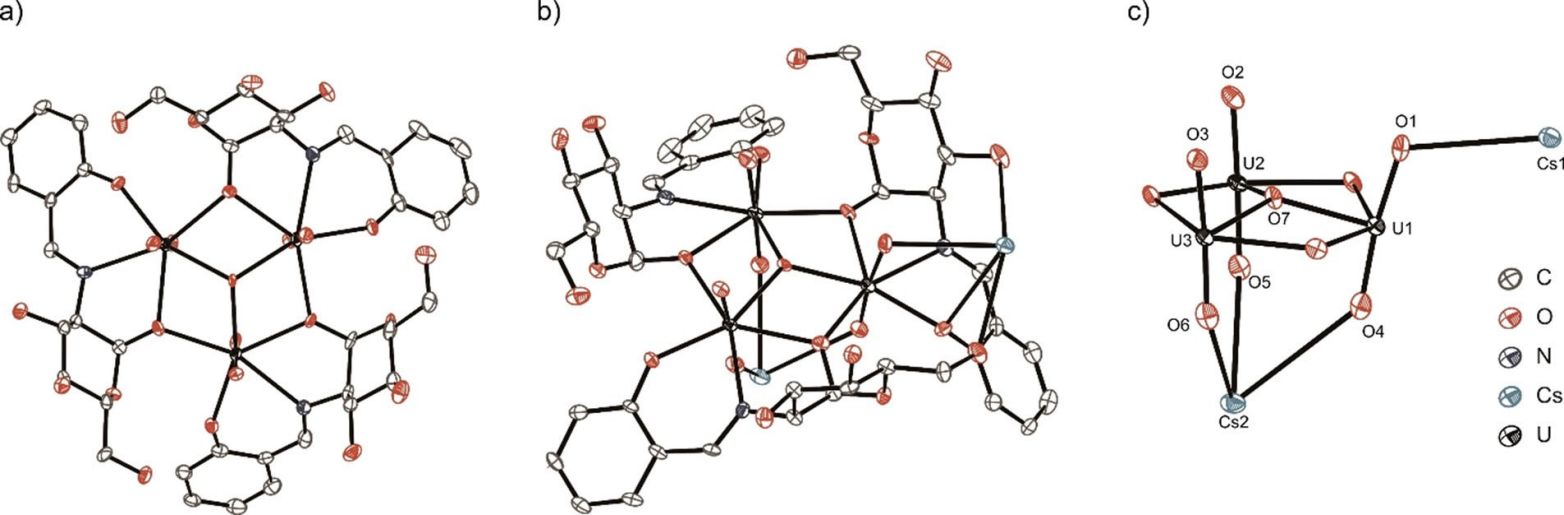
a) Molecular structure of anionic **1** ^2−^ observed in [Cs]_2_ **1** ⋅ 2MeOH (top view); b) molecular structure of [Cs]_2_ **1** ⋅ 2MeOH (side view); c) central trinuclear uranyl core in [Cs]_2_ **1** ⋅ 2MeOH including atom labels; H atoms and solvate molecules are omitted for clarity.

Single crystals of [Cs]_2_ **1** ⋅ 2MeOH, suitable for X‐ray analysis, were obtained on slow evaporation of the filtrate from the above reaction solution (Figure 3). [Cs]_2_ **1** ⋅ 2MeOH crystallizes in the orthorhombic space group *P*2_1_2_1_2_1_, in which each uranyl center is coordinated in its equatorial plane by one (**L^1^**)^2−^. As predicted from the NMR experiments, coordination is seen to occur *via* the phenolate oxygen and the imine nitrogen of the Schiff base moiety as well as by the *α*‐C1‐alcoholate group of the carbohydrate moiety, with the latter forming a *μ_2_*‐O bridge to the neighboring uranyl ion. A *μ_3_*‐oxo ligand is present in the center of the trinuclear complex, which bridges all three uranyl ions. This results in a distorted pentagonal bipyramidal coordination geometry for each uranyl ion. The respective uranyl ions each exhibit O−U−O angles that are slightly distorted from the ideal of 180°, with the average angle being 174.8(3)°; the average U−O bond length is 1.807(9) Å (Table S2).^[18]^ In addition, the three pentagonal bipyramidal U(VI) coordination geometries are slightly tilted towards each other, resulting in the uranyl oxygens (O1‐O6) defining the frustum of a cone. The central *μ_3_*‐oxo ligand (O7) is positioned 0.392 Å above the center of the (UO_2_)_3_‐plane towards the upper rim of the frustum (*i. e*. towards O1‐O3). The three coordinated Schiff base ligands are “fanned out” along the complex's long axis with the three carbohydrate moieties pointing towards O1‐O3, while the three aromatic moieties point in the opposite direction towards O4‐O6.

The negative charge on the trinuclear **1** ^2−^ complex is compensated by the coordination of two Cs^+^ ions. Cs1 is bound to the uranyl oxygen O1 of **1** ^2−^ as well as to a carbohydrate hydroxyl group and the phenolate oxygen of one (**L^1^**)^2−^ together with a further carbohydrate hydroxyl group from a second (**L^1^**)^2−^ moiety. Furthermore, coordination also occurs with O2 and O3 from a neighboring **1** ^2−^ complex and another carbohydrate hydroxyl group of a second neighboring **1** ^2−^ unit, thus resulting in the bridging of three uranyl complex units in total.

**Figure 4:**
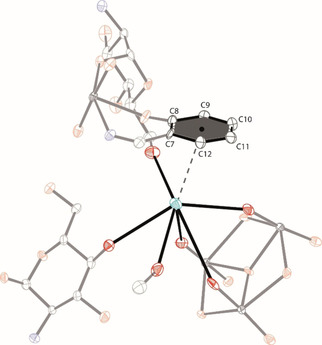
Cation‐π‐interactions between Cs2 and a neighboring aromatic moiety in [Cs]_2_ **1** ⋅ 2MeOH.

Likewise Cs2 also bridges three **1** ^2−^ units. It is bound to the first unit by the uranyl oxygens O4‐O6 (Figure 3c), while the second unit coordinates again by a carbohydrate hydroxyl group. The third **1** ^2−^ unit also binds to Cs2 by a carbohydrate hydroxyl group in addition to one phenolate moiety, which also shows a π‐interaction towards Cs2 (Figure 4). The observed Cs‐plane distance of 3.427(7) Å is well within the range of similar Cs‐π‐interactions of previously reported complexes.^[22]^ Lastly, one molecule of methanol is also bound to Cs2, rounding out the coordination sphere. Overall the coordination of two Cs^+^ ions results in the bridging of individual trinuclear **1** ^2−^ units to form a three dimensional network in the solid state (Figure S26).^[18]^ As mentioned above, the oligomerization behavior of uranyl ions is in general well understood, with a tendency towards more oligonuclear species being formed as the pH is increased.[^4g^, ^4h^, ^4i^, ^4j^] While the speciation of uranyl ions in organic solvents (including methanol) is far less well established than in aqueous solution, mass spectrometric investigations by Jaisen *et al*.^[23]^ and Zhang *et al*.^[24]^ of uranyl ions in methanol did reveal the presence of several species incorporating aqua and/or hydroxyl ligands. These included [UO_2_(CH_3_OH)(OH)]^+^, [UO_2_(CH_3_OH)(H_2_O)(OH)]^+^, [(UO_2_)(CH_3_OH)_2_(OH)]^+^ and [UO_2_(CH_3_OH)(H_2_O)_2_(OH)]^+^. Based on this, the presence of one or more of such aqua/hydroxyl species seems most likely to be the origin of the observed central oxo ligand that occurs in **1** ^2−^.

Further, it is noted that while uranyl is also known to activate molecular oxygen, such activation usually results in the formation of peroxo‐bridged complexes.^[25]^ Thus, we postulate that complex formation of **1** ^2−^ likely proceeds via deprotonation of an aqua or hydroxyl ligand bound to uranyl ion. In this context, our attempts to isolate an oxo‐free uranyl complex of (**L^1^**)^2−^ in the present study proved unsuccessful, even when the reactions were performed under rigorous conditions such as in a nitrogen atmosphere and using carefully dehydrated reagents and dried solvents. In several experiments of this type we were only able to isolate [Cs]_2_ **1** as the product (Figures S5, S6).^[18]^

While this result could reflect the presence of trace amounts of water remaining after the dehydration/drying processes, hexoses are known to readily dehydrate, forming a series of anhydro sugars,^[26]^ which could also provide a source of water and consequentially, the appearance of the *μ_3_*‐oxo group in our complexes.

**Figure 5:**
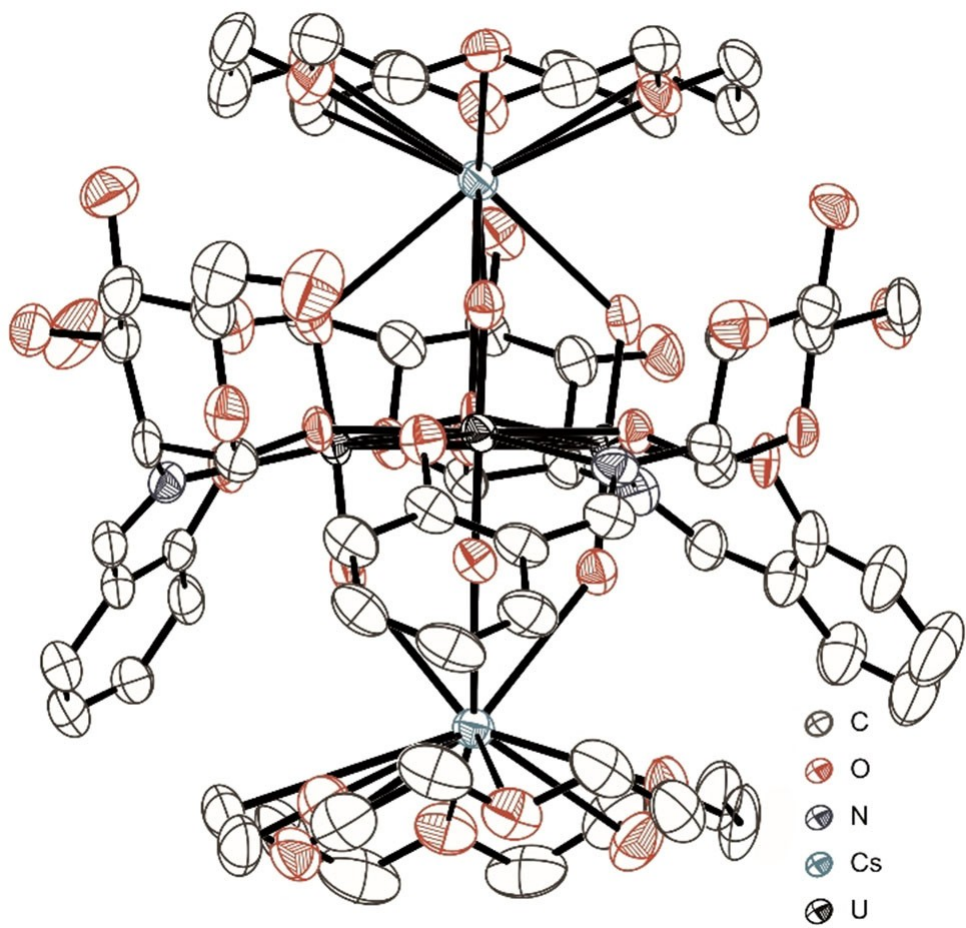
Molecular structure of [Cs(18‐crown‐6)]_2_ **1** in 2([Cs(18‐crown‐6)]_2_ **1**) ⋅ 9DMF ⋅ 2H_2_O, H atoms and solvate molecules are omitted for clarity.

In an extension of the above, [Cs]_2_ **1** was recrystallized from DMF in the presence of 18‐crown‐6 to yield single crystals of 2([Cs(18‐crown‐6)]_2_ **1**) ⋅ 9DMF ⋅ 4H_2_O that were suitable for X‐ray analysis. Likewise the analogous Rb complex [Rb(18‐crown‐6)]_2_ **1** ⋅ 3DMF ⋅ 2H_2_O and the related complex [Cs(18‐crown‐6)]_2_[(UO_2_)_3_(*μ_3_*‐O)(**L^2^**)_3_] ⋅ 4DMF ⋅ H_2_O ([Cs(18‐crown‐6)]_2_ **2** ⋅ 4DMF ⋅ H_2_O) were also obtained using related synthetic procedures.^[18]^ In addition [K(18‐crown‐6)]_2_ **1** was synthesized but no suitable crystals for X‐ray analysis were able to be obtained. Contrary to the Cs^+^ coordination observed in [Cs]_2_ **1** ⋅ 2MeOH, the alkali metal ions present in these complexes are positioned above and below the center of the trinuclear uranyl unit, with an M1‐O7‐M2 angle of 177.2(4)° in 2([Cs(18‐crown‐6)]_2_ **1**) ⋅ 9DMF ⋅ 4H_2_O, 177.9(4)° in [Rb(18‐crown‐6)]_2_ **1** ⋅ 3DMF ⋅ 2H_2_O and 178.43(19)° in ([Cs(18‐crown‐6)]_2_ **2** ⋅ 4DMF ⋅ H_2_O) respectively (with M=Rb, Cs, Figure 5 for Cs‐salt). Each alkali metal center is coordinated in a *κ*_3_‐fashion by a [(UO_2_)_3_(*μ_3_*‐O)(**L^n^**)_3_]^2−^ (n=1, 2) dianion via its respective uranyl oxygens (O1‐O3 for M1, O4‐O6 for M2) as well as by *κ*_6_‐coordination of an 18‐crown‐6 macrocycle. Reflecting the cone shape of the uranyl centers and the off‐center position of the *μ_3_*‐oxo (O7) ligand, a significantly shorter M1‐O7 bond (3.383(7) Å) is present in 2([Cs(18‐crown‐6)]_2_ **1**) ⋅ 9DMF ⋅ 4H_2_O when compared to the M2−O7 bond (4.310(7) Å, Table S2).^[18]^ Unlike [Cs]_2_ **1** ⋅ 2MeOH, which forms a three dimensional network in the solid state, 2([Cs(18‐crown‐6)]_2_ **1**) ⋅ 9DMF ⋅ 4H_2_O, [Rb(18‐crown‐6)]_2_ **1** ⋅ 3DMF ⋅ 2H_2_O and ([Cs(18‐crown‐6)]_2_ **2** ⋅ 4DMF ⋅ H_2_O crystallize as discrete units of the type [M(18‐crown‐6)]_2_[(UO_2_)_3_(*μ_3_*‐O)(**L**)_3_] (M=Rb, Cs for **1** ^2−^, M=Cs for **2** ^2−^).

**Figure 6:**
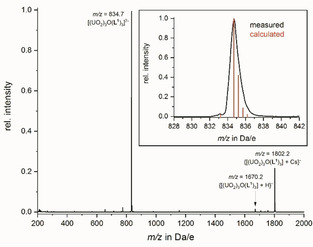
ESI^−^ spectrum of [Cs(18‐crown‐6)]_2_ **1**, including an expanded view of the peak *m/z*=834.7 Da/e with the isotope pattern for **1** ^2−^ superimposed in red.

In an attempt to verify the presence of the trinuclear uranyl complex in solution, ESI‐MS were recorded of the isolated compounds dissolved in methanol. The spectra of [M(18‐crown‐6]_2_ **1** (M=K, Rb; Cs) all display dominant peaks attributed to the free **1** ^2−^ dianion (Figure 6 and Figure S19–S20),^[18]^ in addition to peaks for the related {**1**+H}^−^ and {**1**+M}^−^ ions. In positive ionization mode we observed peaks attributed to the alkali metal crown ether complexes ([M(18‐crown‐6)]^+^ with M=K, Rb, Cs respectively, see Figure S22–24).^[18]^ Likewise the spectrum of [Cs(18‐crown‐6)]_2_ **2** shows the respective **2** ^2−^ dianion peak and the protonated {**2**+H}^−^ peak (Figure S21),^[18]^ although in positive ionization mode the peak for [Cs(18‐crown‐6)]^+^ is absent (see Figure S25).^[18]^ This clearly shows the presence of both **1** ^2−^ and **2** ^2−^ in solution. From the above results combined with those from UV/Vis and NMR (Job plot) experiments we conclude that both H_2_ **L^1^** and H_2_ **L^2^** form exclusively trinuclear uranyl complexes of type [(UO_2_)_3_(*μ_3_*‐O)(**L^n^**)_3_]^2−^ (n=1, 2) in solution. In subsequent experiments we also investigated the coordination behavior of H_2_ **L^1^** towards UO_2_^2+^ in the absence of a base. Upon reacting UO_2_(NO_3_)_2_ ⋅ 6H_2_O with H_2_ **L^1^** in methanol, we observed partial ligand hydrolysis.

**Figure 7:**
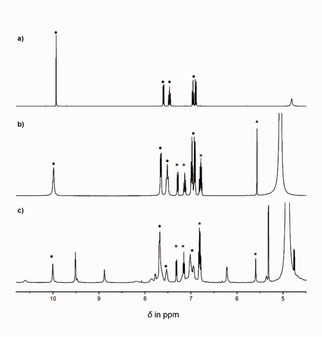
^1^H‐NMR (500 MHz) spectra of a) Salicylic aldehyde; b) Salicylic aldehyde and UO_2_(NO_3_)_2_ (M : L=1 : 1); c) Reaction mixture of H_2_ **L^1^** and UO_2_(NO_3_)_2_ (*c*(UO_2_(NO_3_)_2_)=*c*(H_2_ **L^1^**)=67 mM) in MeOD‐*d*_4_; resonances attributed to unbound salicylic aldehyde are marked with “⋅”, while resonances attributed to metal coordinated salicylic aldehyde are marked with “*”.

**Scheme 3:**
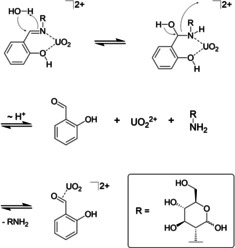
Postulated mechanism for the Lewis acid induced hydrolysis of H_2_ **L^1^** by uranyl ions in absence of a base.

The ^1^H‐NMR spectra of the reaction mixture (Figure 7c) shows several sets of resonances of which two can be identified as being due to the presence of both unbound and coordinated salicylic aldehyde (Figure 7a, b; resonances attributed to unbound salicylic aldehyde are marked with “⋅”, resonances attributed to metal coordinated salicylic aldehyde are marked with “*”). The Lewis acid induced hydrolysis of metal‐bound Schiff bases is well documented^[27]^ and usually postulated to occur *via* a “backside” nucleophilic attack of a water molecule on the imine bond. For uranyl we postulate an analogous hydrolysis mechanism similar to that proposed by Sukanja *et al*. for a ruthenium Schiff base complex.^[27e]^ Following the coordination of the uranyl ion by the ligand's imine nitrogen, electron density of the C=N bond is donated to the metal center, opening up the imine bond for a nucleophilic attack by a water molecule to form the corresponding α‐hydroxyl amine. After proton rearrangement, the C−N bond is broken, forming the free aldehyde and amine (Scheme 3).

Interestingly, in the NMR spectrum for the coordinated uranyl salicylic aldehyde complex (Figure 7b) we observe an upfield shift for all proton resonances (resonances marked with “*”). This includes a very large shift of the aldehyde proton resonance from *δ*(CHO)=9.93 ppm for the unbound aldehyde to *δ*(CHO)=5.57 ppm when metal bound (Figure 7a, b). Likewise, the ^13^C‐NMR spectrum shows an upfield shift from *δ*(CHO)=197 ppm to *δ*(CHO)=102 ppm (Figure S18).^[18]^ Similar upfield shifts have been reported for other metal complexes of benzylic aldehydes^[28]^ as well as related thioaldehydes^[29]^ and silaaldehydes^[30]^ and can be attributed to *η* ^2^‐coordination of the metal centers via the respective C−O double bonds.

**Figure 8:**
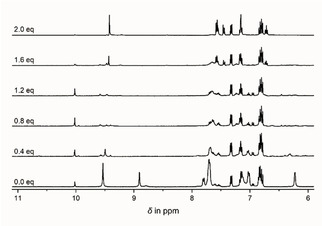
Excerpts of the ^1^H‐NMR of the reaction mixture of H_2_ **L^1^** and UO_2_(NO_3_)_2_ (*c*(UO_2_(NO_3_)_2_=*c*(H_2_ **L^1^**)=10 mM) in MeOD‐*d*_4_ upon the addition of sodium acetate. NaOAc stoichiometry is marked on the left‐hand side.

**Scheme 4:**
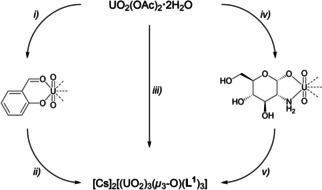
*In situ* assembly of **1**; i) Salicylic aldehyde, MeOH, rt, 2 h; ii) 1. Glucosamine hydrochloride, rt, 2 h, then Cs_2_CO_3_, rt, overnight; iii) 1. H_2_ **L^1^**, MeOH, rt, 2 h, then Cs_2_CO_3_, rt, overnight; iv) Glucosamine hydrochloride, MeOH, rt, 2 h; v) 1. Salicylic aldehyde, rt, 2 h, then Cs_2_CO_3_, rt, overnight.

Following our initial NMR investigation, sodium acetate was added to a freshly prepared solution of H_2_ **L^1^** and UO_2_(NO_3_)_2_ ⋅ 6H_2_O (M : L=1 : 1) in MeOD‐*d_4_*. During the stepwise addition of the acetate, the disappearance of unbound salicylic aldehyde was observed, clearly identified via the progressive loss of the CHO resonance (Figure 8). Simultaneously, a new set of resonances arose that correspond to the formation of **1** ^2−^. Thus, it appears that both the Schiff base ligands as well as the trinuclear complexes are readily formed *in situ* with little regard to reaction management. In further reactions we initially stirred uranyl acetate with salicylic aldehyde to first form the uranyl aldehyde complex *in situ*.^[31]^ Following subsequent addition of glucosamine and cesium carbonate, [Cs]_2_ **1** was the only product able to be isolated. The v*ice versa* procedure of first stirring uranyl acetate with glucosamine and then adding the aldehyde, also yielded [Cs]_2_ **1** as the sole product (Scheme 4).

## Conclusion

The synthesis and characterization of the new solvated (solv=MeOH, H_2_O, DMF) trinuclear uranyl complexes [Cs]_2_ **1 ⋅** solv, [M(18‐crown‐6)_2_ **1 ⋅** solv (M=K, Rb, Cs) and [Cs(18‐crown‐6)]_2_ **2** are reported. The coordination behavior of the 2‐hydroxyaryl glucosamine Schiff bases H_2_ **L^1^** and H_2_ **L^2^** were thoroughly investigated employing several spectroscopic techniques and showed the exclusive formation of the trinuclear dianionic [(UO_2_)_3_(*μ_3_*‐O)(**L^n^**)_3_]^2−^ (n=1, 2) unit. Single crystal X‐ray analysis revealed that the coordination of the alkali metal ion occurs via three uranyl oxygen atoms. The ease of access to Schiff bases of the present type due to the wide range of available 2‐hydroxyaryl aldehydes and suitable functionalized carbohydrate derivatives, coupled with use of the synthetic strategies developed in the present study, opens a wide range of possibilities for the further development of new trinuclear uranyl chemistry and additionally is of importance for the understanding of migration and interaction of uranyl‐ions in the environment.

## Conflict of interest

The authors declare no conflict of interest.

## Supporting information

As a service to our authors and readers, this journal provides supporting information supplied by the authors. Such materials are peer reviewed and may be re‐organized for online delivery, but are not copy‐edited or typeset. Technical support issues arising from supporting information (other than missing files) should be addressed to the authors.

Supplementary
