# Supplementary material for: Saccharified Uranyl Ions: Self‐Assembly of UO2 2+ into Trinuclear Anionic Complexes by the Coordination of Glucosamine‐Derived Schiff Bases
Source: Chemistry. 2021 May 21;27(33):8484–91. doi: 10.1002/chem.202100546 (PMC8252515; doi:10.1002/chem.202100546)
Supplement: Supplementary file 1 — Supplementary [file CHEM-27-8484-s001.pdf]

Chemistry–A European Journal

Supporting Information

Saccharified Uranyl Ions: Self-Assembly of UO_2^{2+} into Trinuclear Anionic Complexes by the Coordination of Glucosamine-Derived Schiff Bases

Gerrit Schaper, Marco Wenzel, Felix Hennersdorf, Leonard F. Lindoy, and Jan J. Weigand*

Table of Contents

Table of Contents 2
S1. General remarks. 2
S2. Syntheses and Spectroscopic Data 4
S2.1 Preparation of 2-salicylidene glucosamine (H_2L^1) 4
S2.2 Preparation of 2-(2-Hydroxy-1-naphtylidene) glucosamine (H_2L^2) 6
S2.3 Preparation of $[Cs]_2[(UO_2)_3(\mu_3\text{-}O)(L^1)_3]$ ($[Cs]_2$1) 8
S2.4 Alternative synthetic approaches for $[Cs]_2$1 8
S2.5 Preparation of $[Cs(18\text{-crown-}6)]_2[(UO_2)_3(\mu_3\text{-}O)(L^1)_3]$ ($[Cs(18\text{-crown-}6)]_2$1) 10
S2.6 Preparation of $[Rb(18\text{-crown-}6)]_2[(UO_2)_3(\mu_3\text{-}O)(L^1)_3]$•0.5DMF ($[Rb(18\text{-crown-}6)]_2$1•0.5DMF) 12
S2.7 Preparation of $[K(18\text{-crown-}6)]_2[(UO_2)_3(\mu_3\text{-}O)(L^1)_3]$ ($[K(18\text{-crown-}6)]_2$1) 13
S2.8 Preparation of $[Cs(18\text{-crown-}6)]_2[(UO_2)_3(\mu_3\text{-}O)(L^2)_3]$ ($[Cs(18\text{-crown-}6)]_2$2) 15
S3. Additional spectroscopic data 16
S3.1 Job plot experiments of UO_2^{2+} and H_2L^2 16
S3.2 Lewis acid induced hydrolysis of H_2L^1 18
S3.3 MS-spectra 19
S4. Crystallographic Details 22
S4.1 Crystallographic Refinement 22
References 25

S1. General remarks.

General Considerations: All reported compounds were fully characterized by multinuclear NMR spectroscopy, IR- and Raman spectroscopy and elemental analysis as well as single crystal X-ray crystallography where applicable. Manipulations were performed under ambient conditions or, when stated, in a Glovebox MB Unilab or using Schlenk techniques under an atmosphere of purified Argon. Dry, oxygen-free solvents (MeOH (distilled from Mg), diethyl ether (distilled from potassium)) were employed. All distilled solvents were stored over molecular sieve (3 Å: MeOH, 4 Å: Et_2O). Water content of solvents was monitored via coulometric Karl-Fischer-titration using an Aqua 40.00 titrator by Elektroktrochemie Halle GmbH and is quoted in ppm (MeOH: 4-6 ppm, Et_2O: 5-10 ppm). Deuterated dimethyl sulfoxide (DMSO-d_6, 99.9%) was purchased from Sigma Aldrich, deuterated methanol (MeOH-d_4, 99.8%) was purchased from Deutero and used without further purification. Anhydrous DMSO-d_6 was prepared from freshly opened DMSO-d_6, degassed and stored over molecular sieve (3 Å) under a nitrogen atmosphere. All glassware were oven-dried at 160 °C prior to use.

Compounds H_2**L¹** [1] and H_2**L²** [2], anhydrous uranyl acetate[3] and anhydrous cesium acetate[4] were prepared according to procedures given in the literature. Glucosamine hydrochloride 98% was purchased from TCI Japan, while salicylic aldehyde reagent grade 98%, cesium carbonate ReagentPlus 99% and 18-crown-6 99% were purchased from Sigma Aldrich, sodium bicarbonate 99% was purchased from Grüssing, sodium acetate trihydrate purissimus was purchased from Isocommerz, potassium acetate 99%, rubidium acetate 99.8%, cesium acetate 99% and 2-hydroxy-1-naphthaldehyde 98% were purchased from Alfa Aesar, triethylamine 99.5% was purchased from Roth and used without further purification. NMR spectra were measured on a Bruker AVANCE III HD Nanobay (^{1}H (400.13 MHz), ^{13}C (100.61 MHz)) 400 MHz UltraSield or on a Bruker AVANCE III HDX, 500 MHz Ascend (^{1}H (500.13 MHz), ^{13}C (125.75 MHz) instrument). All ^{13}C NMR spectra were exclusively recorded with composite pulse decoupling. Reported numbers assigning atoms in the ^{13}C spectra were indirectly deduced from the cross-peaks in 2D correlation experiments (HMBC, HSQC). Chemical shifts were referenced to δ_{TMS} = 0.00 ppm (^{1}H, ^{13}C) and are reported in ppm. Coupling constants (*J*) are reported in Hz. NMR-titration experiments were performed via the method of continuous variation (Job's method) as a batch titration. H_2**L** and uranyl acetate ($UO_2(OAc)_2{\cdot}2H_2O$) stock solutions with $c_0(H_2\mathbf{L}) = c_0(UO_2(OAc)_2{\cdot}2H_2O)$ = 0.01 M were prepared in DMSO-*d*$_6$. Isochoric samples of V = 0.5 ml with varying molar ratio of UO_2^{2+} were prepared from $x(UO_2^{2+}) = 0$ to $x(UO_2^{2+}) = 0.9$ and shaken at room temperature overnight. ^{1}H-NMR were recorded on a Bruker AVANCE III HD Nanobay 400 MHz UltraShield instrument at room temperature. Melting points (m.p.) were recorded on an electrothermal melting point apparatus (Büchi Switzerland, Melting point M-560) in capillaries and are uncorrected. Infrared (IR) and Raman spectra were recorded at ambient temperature using a Bruker Vertex 70 instrument equipped with a RAM II module (Nd-YAG laser, 1064 nm). The Raman intensities are reported in percent relative to the most intense peak and are given in parenthesis. An ATR unit (diamond) was used for recording IR spectra. The intensities are reported relative to the most intense peak and are given in parenthesis using the following abbreviations: vw = very weak, w = weak, m = medium, s = strong, vs = very strong. Elemental analyses were performed on a Vario MICRO cube Elemental Analyzer by Elementar Analysatorsysteme GmbH in CHNS modus. **Note:** In some cases, particularly for ([K(18-crown-6)]$_2$**1**, we obtained comparable low carbon values since the complete combustion, even with the addition of WO_3, is hampered due to the formation of stable carbonates.[5,6] UV/Vis data were recorded on a Perkin Elmer LAMBDA 25 double beam spectrometer at room temperature against blank solvent. UV/Vis-titration experiments were performed via the method of continuous variation (Job's method) as a batch titration. H_2**L** and uranyl acetate ($UO_2(OAc)_2{\cdot}2H_2O$) stock solutions with $c_0(H_2L) = c_0(UO_2(OAc)_2{\cdot}2H_2O)$ = 1.0 mM were prepared in MeOH. Isochoric samples of V = 0.5 ml with varying molar ratio of UO_2^{2+} were prepared from $x(UO_2^{2+}) = 0$ to $x(UO_2^{2+}) = 1.0$ and shaken at room temperature overnight. UV/Vis spectra were recorded at room temperature. MS-data were recorded on a Waters ACQUITY UPLC H-class system consisting of Waters ACQUITY Quaternary Solvent Manager, Waters ACQUITY Sample Manager FTN, Waters ACQUITY Column Manager, Waters ACQUITY PDA detector and a Waters ACQUITY tandem quadrupole detector (TQD) with Zspray ESI/APCI/ESCi ion source. Nitrogen, provided by a CMC instruments membrane nitrogen generator (NGM-11-LC/MS), was used as both the desolvation and the cone gas. For CID experiments, argon was used as the collision gas. A constant flow of 0.200 ml/min of 0.1% formic acid/MeCN (1:1 v/v) was provided by the UPLC solvent pump. Samples were dissolved in MeOH (LC-MS grade) and injected via the direct injection at the TQD. MS-Data were recorded via electrospray ionization (ESI) in both positive and negative mode either in combined flow state (sample flow and LC-flow are combined and injected into the ion source) or in infusion flow state (only the sample flow is injected into the ion source). Unless mentioned otherwise the following settings were used for ionization: *U*(capillary) = 3 kV, *U*(cone) = 30 V, c(extractor) = 1 V, *U*(RF-lense) = 0.1 V, *T*(source) = 150 °C, *T*(desolvation gas) = 500 °C, *Q*(desolvation gas) = 500 l/h, *Q*(cone gas) = 50 l/h, *Q*(collision gas) = 0.1 l/h. The spectrometer was calibrated using sodium iodide (2 µg/µl) / cesium iodide (50 ng/µl) in 50/50 iso-propanol/water solution via Acquity Intellistart software. Data is accurate up to 0.2 Da.

S2. Syntheses and Spectroscopic Data

S2.1 Preparation of 2-salicylidene glucosamine (H_2**L**^{1})

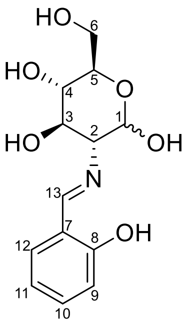

H_2**L**^{1} was synthesized according to the literature.[1] Glucosamine hydrochloride (5.07 g, 23.5 mmol, 1.0 eq.) and sodium bicarbonate (3.21 g, 38.3 mmol, 1.6 eq.) were dissolved in water (50 ml) and salicylaldehyde (3.20 ml, 3.80 g, 31.2 mmol, 1.3 eq.) was added. The reaction solution began to turn yellow immediately. After stirring the solution at room temperature for 15 h, the yellow precipitate was filtered off and washed with cold water (2 x 10 ml) and diethylether (5 x 20 ml). The target compound was obtained by recrystallization from hot methanol as a yellow solid.

Yield: 4.57 g (69%); **m.p.:** 182 °C; **Raman** (100 mW, 1000 scans, 300 K, [cm^{-1}]) :3057 (16), 2943 (19), 2895 (20), 1630 (100), 1581 (81), 1463 (87), 1355 (89), 1317 (85), 1287 (84), 1239 (84), 1157 (83), 1069 (83), 1027 (84), 1008 (84), 902 (85), 781 (92), 626 (86), 562 (89), 524 (89), 464 (89), 428 (90), 193 (85); **IR** (ATR, 300 K, [cm^{-1}]):3371 (w), 2964 (w), 2936 (w), 2881 (w), 2733 (w), 1627 (s), 1518 (w), 1490 (m), 1449 (w), 1431 (w), 1405 (m), 1354 (m), 1338 (m), 1304 (w), 1278 (m), 1251 (m), 1226 (m), 1203 (m), 1153 (s), 1091 (vs), 1024 (vs), 1006 (vs), 916 (m), 894 (s), 864 (m), 833 (m), 786 (m), 766 (vs), 739 (s), 713 (m), 591 (vs), 575 (vs), 560 (vs), 550 (vs), 539 (vs), 525 (vs), 497 (s), 455 (s), 410 (s) **^{1}H-NMR** (500 MHz, DMSO-d_6, 300 K, in ppm): δ = (Two sets of resonances were identified and attributed to two anomers, α/β = 0.45) 2.85-2.88 (1H, m, β-2-H), 3.14-3.22 (2H, m, β-4-H, β-5-H), 3.26-3.29 (2H, m, α-2-H, α-4-H), 3.42-3.56 (3H, m, α-6-H, β-3-H, β-6-H), 3.66-3.74 (4H, m, α-3-H, α-5-H, α-6-H', β-6-H'), 4.47 (1H, s(br.), v1/2≈ 15 Hz, α-6-OH), 4.57 (1H, s(br.), v1/2≈ 13 Hz, β-6-OH), 4.71 (1H, pseudo-t, $^3J_{1H,1OH}$ = 6.4 Hz, $^3J_{1H,2H}$ = 7.4 Hz, β-1-H), 4.95-5.01 (3H, m, α-3-OH, α-4-OH, β-4-OH), 5.04 (1H, pseudo-t, $^3J_{1H,1OH}$ = 4.2 Hz, $^3J_{1H,2H}$ = 3.0 Hz, α-1-H), 5.10 (1H, d, $^3J_{3H,3OH}$ = 5.2 Hz, β-3-OH), 6.57 (1H, d, $^3J_{1H,1OH}$ = 4.2 Hz, α-1-OH), 6.76 (1H, d, $^3J_{1H,1OH}$ = 6.4 Hz, β-1-OH), 6.83-6.92 (4H, m, α-9-H, α-11-H, β-9-H, β-11-H), 7.29-7.35 (2H, m, α-10-H, β-10-H), 7.42 (1H, dd, $^3J_{11H,12H}$ = 8.0 Hz, $^4J_{10H,12H}$ = 1.4 Hz, α-12-H), 7.48 (1H, dd, $^3J_{11H,12H}$ = 7.8 Hz, $^4J_{10H,12H}$ = 1.4 Hz, β-12-H), 8.41 (1H, s, β-13-H), 8.48 (1H, s, α-13-H), 13.36 (1H, s, β-8-OH), 14.07 (1H, s, α-8-OH); **^{13}C{^{1}H}-NMR** (125 MHz, DMSO-d_6, 300 K, in oom): δ = 6.11, (1C, s, β-6-C), 6.16 (1C, s, α-6-C), 70.15 (1C, s, β-4-C), 70.58 (1C, s, α-4-C), 70.91 (1C, s, α-3-C), 72.37 (2C, s, α-5-C, β-5-C), 74.61 (1C, s, β-3-C), 76.46 (1C, s, β-2-C), 76.86 (1C, s, α-4-C), 92.02 (1C, s, α-1-C), 95.24 (1C, s, β-1-C), 116.32 (1C, s, β-11-C), 116.87 (1C, s, α-11-C), 117.87 (1C, s, α-9-C), 118.54 (1C, s, 7-α-C), 118.56 (1C, s, β-9-C), 118.86 (1C, s, β-7-C), 131.66 (1C, s, β-12-C) 131.73 (1C, s, α-12-C), 132.19 (1C, s, β-10-C), 132.35 (1C, s, α-4-C), 160.46 (1C, s, β-8-C), 161.93 (1C, s, α-8-C), 166.52 (1C, s, α-13-C), 166.70 (1C, s, β-13-C); **ESI-MS:** m/z [Da/e] = 284.2 ([H_2**L**^{1}+H]$^+$); **Elemental Analysis:** calculated for $C_{13}H_{17}NO_6$ C: 55.12%, N: 4.94%, H: 6.05%, found C: 54.82%, N: 4.98%, H: 5.78%

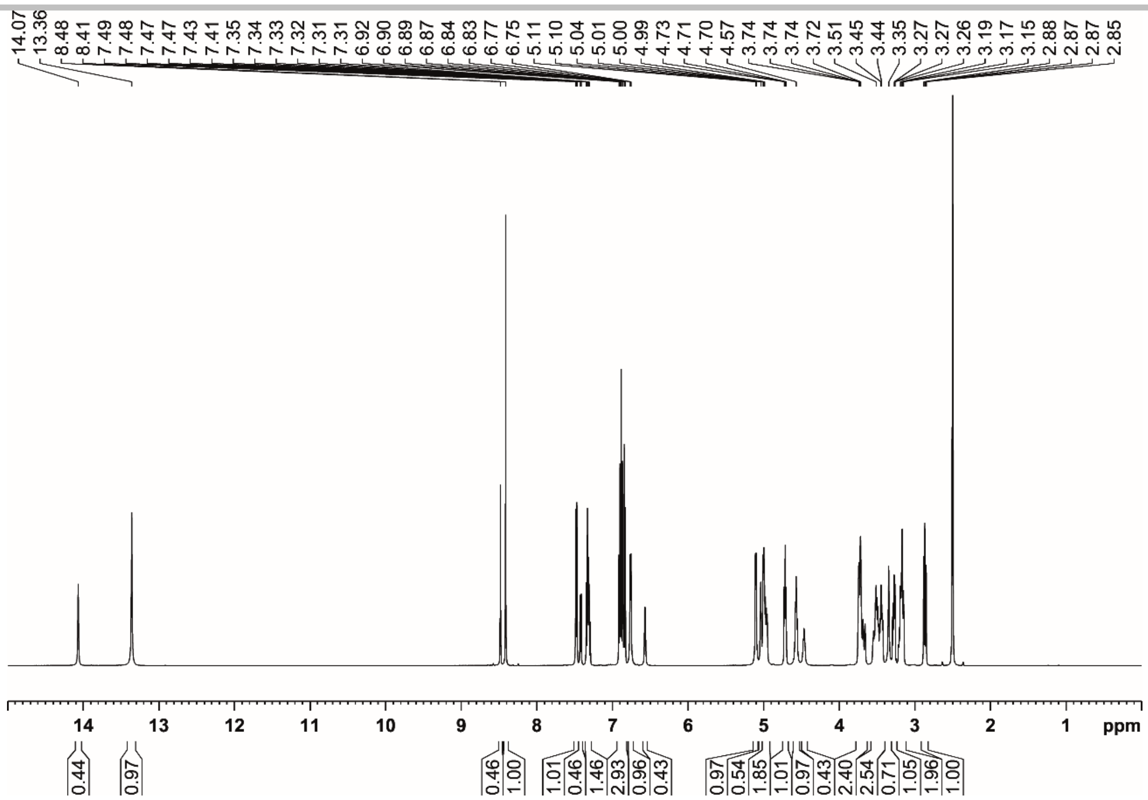

Figure S1: ^{1}H-NMR spectrum of H_2**L**^{1}.

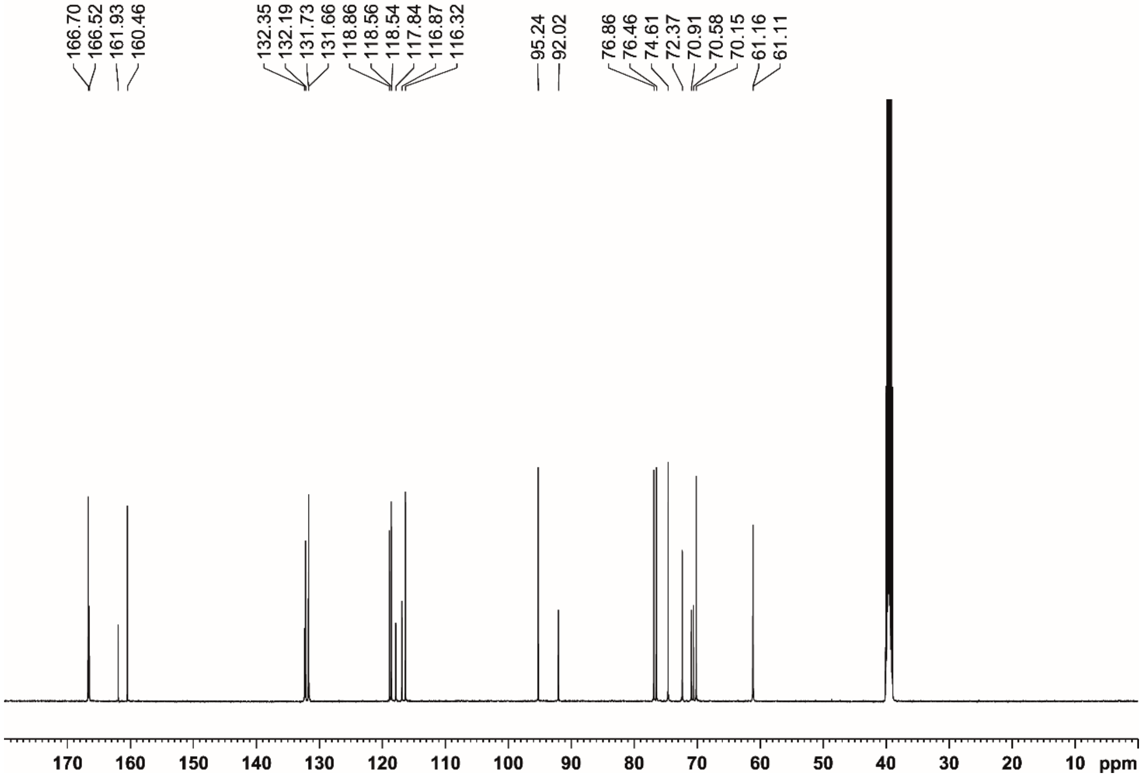

Figure S2: $^{13}C\{^{1}H\}$-NMR spectrum of H_2**L**^{1}.

S2.2 Preparation of 2-(2-Hydroxy-1-naphtylidene) glucosamine (H_2L^2)

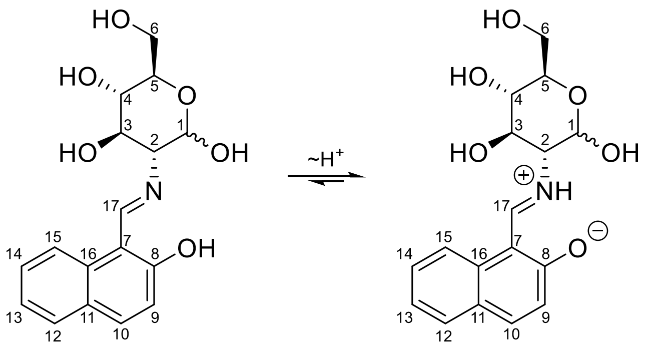

H_2L^2 was synthesized according to the literature.[2] Glucosamine hydrochloride (1.05 g, 4.87 mmol, 1 eq.) and sodium acetate trihydrate (1.89 g, 13.9 mmol, 2.9 eq.) were dissolved in water (10 ml) and 2-hydroxy-1-naphthaldehyde (1.08 g, 6.27 mmol, 1.3 eq.) in methanol (40 ml) was added. After stirring at room temperature for 22 h in the dark, the precipitate that had formed was filtered off and washed with ice water (3 x 5 ml) then diethylether (5 x 10 ml). The target compound was obtained as a crystalline yellow solid following recrystallisation of the initial product from hot methanol. Crystals of α-H_2L^2 suitable for single crystal x-ray analysis were obtained following a further recrystallization from hot methanol.

Yield: 0.84 g (52%); **m.p.:** 188 °C; **Raman** (100 mW, 1000 scans, 300 K, [cm^{-1}]) : 3058 (10), 2962 (7), 2935 (13), 2908 (12), 1636 (15), 1617 (15), 1601 (11), 1547 (34), 1453 (31), 1408 (10), 1392 (9), 1360 (24), 1344 (100), 1305 (11), 1256 (22), 1217 (12), 1170 (10), 1110 (6), 1043 (8), 936 (7), 755 (13), 725 (15), 535 (6), 448 (9), 422 (6), 202 (11), 169 (11); **IR** (ATR, 300 K, [cm^{-1}]): 3470 (vw), 3214 (w), 3027 (w), 2853 (w), 1632 (m), 1615 (m), 1543 (m), 1518 (w), 1504 (w), 1484 (m), 1407 (m), 1358 (m), 1342 (m), 1302 (m), 1262 (m), 1213 (m), 1191 (m), 1131 (m), 1104 (s), 1081 (m), 1052 (s), 1038 (s), 1004 (vs), 966 (m), 933 (m), 870 (s), 830 (s), 781 (s), 739 (vs), 723 (vs), 692 (s), 646 (s), 559 (vs), 517 (s); **^{1}H-NMR** (500 MHz, DMSO-d_6): δ [ppm] = (Two sets of resonances were identified and attributed to two anomers, α/β = 20) 3.17-3.24 (2H, m, α-4-H, β-4-H), 3.28-3.33 (1H, m , β-5-H), 3.49-3.63 (6H, m, α-2-H, α-3-H, α-6-H, β-2-H, β-3-H, β-6-H), 3.67-3.72 (3H, m, α-5-H, α-6-H', β-6-H'), 4.52 (1H, pseudo-t, $^3J_{6H,6OH}$ = 5.8 Hz, $^3J_{6H',6OH}$ = 5.8 Hz, α-6-OH), 4.61 (1H, pseudo-t, $^3J_{6H,6OH}$ = 5.7 Hz, $^3J_{6H',6OH}$ = 5.7 Hz, β-6-OH), 4.75 (1H, pseudo-t, $^3J_{1H,2H}$ = 6.8 Hz, $^3J_{1H,1OH}$ = 7.7 Hz, β-1-H), 5.05 (1H, d, $^3J_{4H,4OH}$ = 5.9 Hz, α-4-OH), 5.20 (1H, pseudo-t, $^3J_{1H,2H}$ = 3.3 Hz, $^3J_{1H,1OH}$ = 4.4 Hz, α-1-H), 5.11 (1H, d, $^3J_{4H,4OH}$ = 5.4 Hz, β-4-OH), 5.32 (1H, d, $^3J_{3H,3OH}$ = 5.8 Hz, α-3-OH), 5.37 (1H, d, $^3J_{3H,3OH}$ = 5.8 Hz, β-3-OH), 6.48 (1H, d, $^3J_{9H,10H}$ = 9.6 Hz, β-9-H), 6.65 (1H, d, $^3J_{9H,10H}$ = 9.5 Hz, α-9-H), 6.81 (1H, d, $^3J_{1H,1OH}$ = 7.7 Hz, β-1-OH), 7.00 (1H, d, $^3J_{1H,1OH}$ = 4.4 Hz, α-1-OH), 7.16 (1H, pseudo-t, $^3J_{12H,13H}$ = 7.7 Hz, $^3J_{13H,14H}$ = 7.0 Hz, α-13-H), 7.23 (1H, *pseudo*-t, $^3J_{12H,13H}$ = 7.8 Hz, $^3J_{13H,14H}$ = 7.1 Hz, β-13-H), 7.41 (1H, *pseudo*-t, $^3J_{13H,14H}$ = 7.0 Hz, $^3J_{14H,15H}$ = 8.4 Hz, α-14-H), 7.46 (1H, *pseudo*-t, $^3J_{13H,14H}$ = 7.1 Hz, $^3J_{14H,15H}$ = 8.5 Hz, β-14-H), 7.59 (1H, d, $^3J_{12H,13H}$ = 7.7 Hz, α-12-H), 7.68 (2H, d, $^3J_{9H,10H}$ = 9.5 Hz, α-10-H, β-12-H), 7.77 (1H, d, $^3J_{9H,10H}$ = 9.6 Hz, β-10-H), 7.97 (1H, d, $^3J_{14H,15H}$ = 8.4 Hz, α-15-H), 8.09 (1H, d, $^3J_{14H,15H}$ = 8.5 Hz, β-15-H), 8.92 (1H, d, $^3J_{17H,NH}$ = 11.5 Hz, α-17-H), 9.06 (1H, d, $^3J_{17H,NH}$ = 7.9 Hz, β-17-H), 13.66 (1H, *pseudo*-t, $^3J_{2H,NH}$ = 8.4 Hz, $^3J_{17H,NH}$ = 11.5 Hz, α-NH), 14.45 (1H, *pseudo*-t, $^3J_{2H,NH}$ = 5.8 Hz, $^3J_{17H,NH}$ = 7.9 Hz, β-NH); **^{13}C{^{1}H}-NMR** (125 MHz, DMSO-d_6, 300 K, in ppm): δ = 48.59 (1C, s, α-2-C), 60.91 (1C, s, α-6-C), 61.01 (1C, s, β-6-C), 65.33 (1C, s, β-2-C), 70.26 (1C, s, β-4-C), 70.49 (1C, s, α-4-C), 71.44 (1C, s, α-3-C), 72.44 (1C, s, α-5-C), 74.21 (1C, s, β-3-C), 76.77 (1C, s, β-5-C), 90.80 (1C, s, α-1-C), 94.84 (1C, s, β-1-C), 105.23 (1C, s, α-7-C), 106.31 (1C, s, β-7-C), 118.08 (1C, s, α-15-C), 118.63 (1C, s, β-15-C), 121.94 (1C, s, α-13-C), 122.37 (1C, s, β-13-C), 124.22 (1C, s, β-11-C), 124.90 (1C, s, α-11-C), 125.56 (1C, s, β-9-C), 126.38 (1C, s, α-9-C), 127.90 (2C, s, α-14-C, β-14-C), 128.87 (2C, s, α-12-C, β-12-C), 133.83 (1C, s, β-16-C), 134.66 (1C, s, α-16-C), 136.44 (1C, s, β-10-C), 137.44 (1C, s, α-10-C), 158.35 (1C, s, α-17-C), 160.35 (1C, s, β-17-C), 174.24 (1C, s, β-8-C), 179.26 (1C, s, α-8-C); **ESI-MS:** m/z [Da/e] = 334.2 ([H_2L^2+H]+); **Elemental Analysis**: calculated for $C_{17}H_{19}NO_6$ C: 61.25%, N: 4.20%, H: 5.75%, found C: 60.85%, N: 4.23%, H: 5.44%

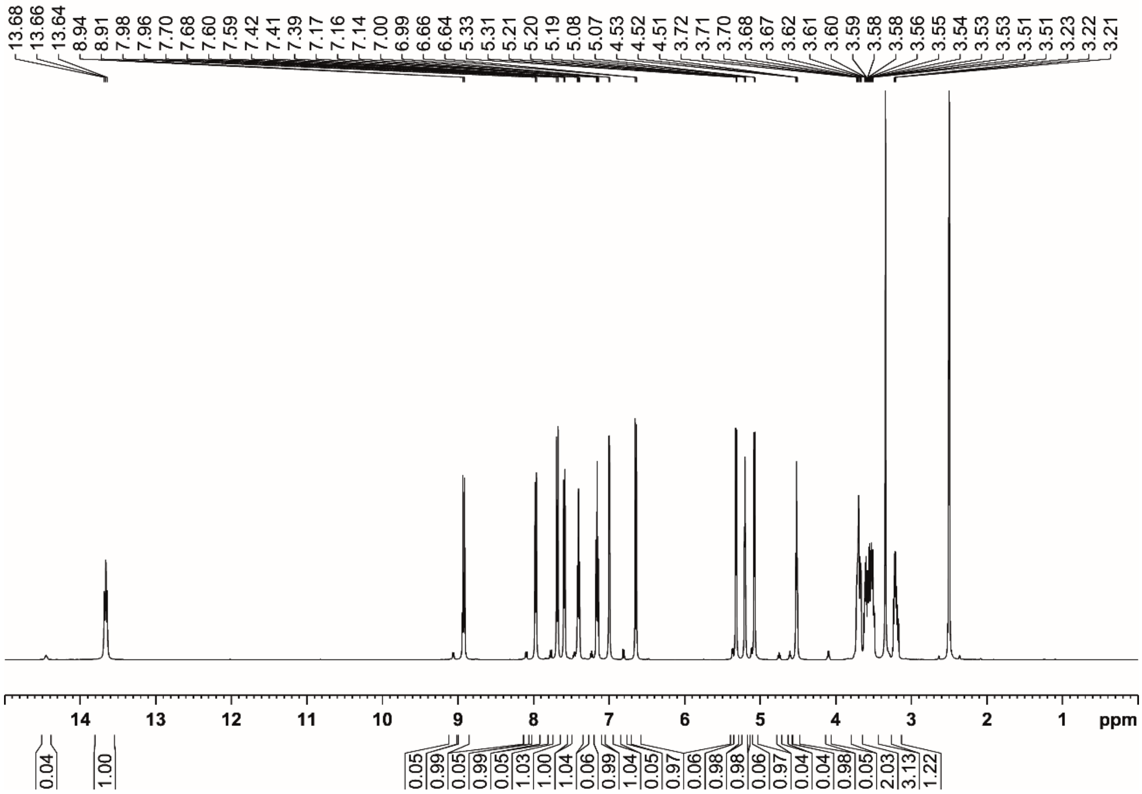

Figure S3: ^{1}H-NMR spectrum of H_2**L²**.

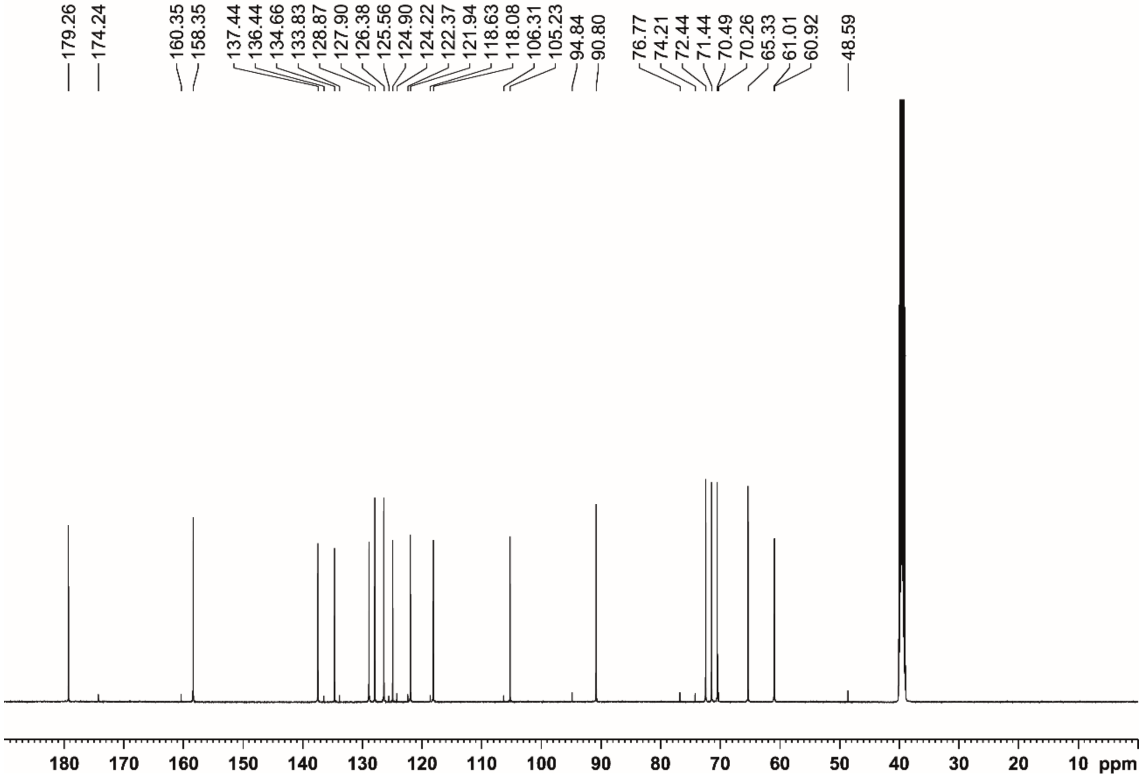

Figure S4: $^{13}C\{^{1}H\}$-NMR spectrum of H_2**L²**.

S2.3 Preparation of $[Cs]_2[(UO_2)_3(\mu_3\text{-}O)(L^1)_3]$ ($[Cs]_2$**1**)

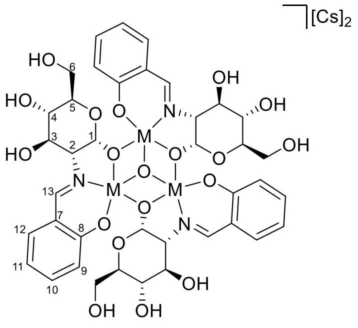

M = UO_2

H_2**L**^{1} (53 mg, 0.19 mmol, 4.5 eq.) was dissolved in MeOH (3 ml). Uranyl acetate dihydrate (53 mg, 0.12 mmol, 3.0 eq.) was dissolved in methanol (4.5 ml) and slowly added to the reaction mixture. After stirring the solution at room temperature for 2 h, a solution of cesium carbonate (27 mg, 0.08 mmol, 2.0 eq.) in methanol (1.5 ml) was added and the reaction mixture was stirred at room temperature for additional 22 h. The yellow precipitate was filtered off, washed with diethylether (3 x 2 ml) and dried in vacuo. The target compound was isolated as a yellow solid. Single crystals suitable for x-ray analysis of $[Cs]_2$**1**·2MeOH were obtained by slow evaporation of a methanol solution of the raw product.

Yield: 68 mg (88%); **m.p.:** 273 °C (decomp.); **Raman** (100 mW, 1000 scans, 300 K, [cm^{-1}]) : 3051 (20), 2886 (30), 1626 (100), 1599 (79), 1551 (99), 1475 (76), 1452 (76), 1330 (94), 1255 (64), 1208 (64), 1159 (62), 1034 (67), 979 (58), 942 (57), 802 (73), 771 (62), 600 (52), 557 (51), 517 (50), 476 (51); **IR** (ATR, 300 K, [cm^{-1}]): 3339 (w), 2883 (vw), 1620 (m), 1597 (w), 1549 (w), 1473 (w), 1449 (w), 1398 (w), 1371 (w), 1328 (w), 1297 (m), 1205 (w), 1130 (m), 1090 (m), 1032 (s), 995 (m), 939 (w), 883 (vs), 864 (m), 846 (s), 813 (w), 793 (m), 761 (s), 740 (m), 686 (m), 657 (m), 610 (m), 575 (m), 554 (m), 515 (s), 494 (vs), 460 (s), 444 (s), 430 (s); **^{1}H-NMR** (500 MHz, DMSO-d_6, 300 K, in ppm): δ = 3.48-3.53 (3H, m, 4-H), 3.90 (3H, *pseudo*-t, $^2J_{6H,6H'}$ = 9.0 Hz, $^3J_{5H,6H}$ = 9.6 Hz, 6-H), 4.06 (3H, *pseudo*-t, $^2J_{6H,6H'}$ = 9.0 Hz, $^3J_{6H',6OH}$ = 9.8 Hz, 6-H'), 4.43-4.48 (3H, m, 3-H), 4.51 (3H, dd, $^3J_{2H,3H}$ = 9.2 Hz, $^3J_{1H,2H}$ = 3.4 Hz, 2-H), 4.83 (3H, d, $^3J_{4H,4OH}$ = 5.7 Hz, 4-OH), 4.95 (3H, *pseudo*-t, $^3J_{4H,5H}$ = 8.7 Hz, 9.6 Hz, $^3J_{5H,6H}$ = 9.6 Hz, 5-H), 5.11 (3H, d, $^3J_{3H,3OH}$ = 5.5 Hz, 3-OH), 6.26 (3H, d, $^3J_{6H',6OH}$ = 9.8 Hz, 6-OH), 6.63 (3H, *pseudo*-t, $^3J_{10H,11H}$ = 6.8 Hz, $^3J_{11H,12H}$ = 7.7 Hz, 11-H), 6.93 (3H, d, $^3J_{1H,2H}$ = 3.4 Hz, 1-H), 7.35 (3H, d, $^3J_{9H,10H}$ = 8.3 Hz 9-H), 7.49-7.52 (3H, m, 10-H), 7.55 (3H, dd, $^3J_{11H,12H}$ = 7.7 Hz, $^4J_{10H,12H}$ = 1.45 Hz, 12-H), 9.24 (3H, s, 13-H); **^{13}C{^{1}H}-NMR** (125 MHz, DMSO-d_6, 300 K, in ppm): δ = 63.40 (3C, s, 6-C), 72.14 (6C, s, 3-C, 5-C), 72.38 (3C, s, 4-C), 83.34 (3C, s, 2-C), 100.25 (3C, s, 1-C), 115.37, (3C, s, 11-C), 120.73 (3C, s, 9-C), 123.79 (3C, s, 7-C), 133.47 (3C, s, 10-C/12-C), 133.56 (3C, s, 10-C/12-C), 166.66 (3C, s, 13-C), 170.14 (3C, s, 8-C); **Elemental analysis:** calculated for $[Cs]_2$**1** ($C_{39}H_{45}N_3O_{25}U_3$) C: 24.20%, H: 2.34%, N: 2.17%, found C: 23.79%, H: 2.17%, N: 2.30%

S2.4 Alternative synthetic approaches for $[Cs]_2$1

Version 2:

2-Salicylidene glucosamine (56 mg, 0.20 mmol, 3.0 eq.) was dissolved in methanol (5 ml) and triethylamine (55.5 µl, 0.40 mmol, 6.0 eq.) was added. Uranyl nitrate hexahydrate (98 mg, 0.20 mmol, 3.0 eq.) was dissolved in methanol (2 ml) and slowly added to the reaction mixture. After stirring the mixture at room temperature for 2 h, a solution of cesium carbonate (45 mg, 0.14 mmol, 2.1 eq.) in methanol (1.5 ml) was added and the reaction mixture was stirred at room temperature for additional 72 h. The yellow precipitate was filtered off, washed with diethylether (3 x 2 ml) and dried in vacuo. The target compound was isolated as a yellow solid.

Yield: 127 mg (98%)

Version 3:

Uranyl acetate (100 mg, 236 µmol, 3.0 eq.) was dissolved in methanol (5 ml). Salicylic aldehyde (25 µl, 29 mg, 239 µmol, 3.0 eq.) was added and the reaction mixture was stirred at room temperature for 2 h. Glucosamine hydrochloride (51 mg, 236 µmol, 3.0 eq.) was dissolved in water (1 ml) and added to the reaction mixture. After stirring at room temperature for 1.5 h, cesium carbonate (77 mg, 236 µmol, 3.0 eq.) dissolved in methanol (2 ml) was added and the reaction mixture was stirred at room temperature for 18.5 h. The yellow precipitate was filtered off, washed with diethyl ether (3 x 2 ml) and dried in vacuo. The target compound was isolated as a yellow solid.

Yield: 138 mg (91%)

Version 4:

Uranyl acetate (60 mg, 140 µmol, 3.0 eq.) was dissolved in methanol (3 ml). Glucosamine hydrochloride (31 mg, 140 µmol, 3.0 eq.) dissolved in water (0.6 ml) was added. The reaction mixture was stirred at room temperature for 2 h. Salicylic aldehyde (15 µl, 18 mg, 140 µmol, 3.0 eq.) was then added and the reaction mixture was stirred for an additional 2 h. Cesium carbonate (47 mg, 140 µmol, 3.0 eq.) dissolved in methanol (1.5 ml) was added and the reaction mixture stirred for a further 22 h. The yellow precipitate that formed was filtered off, washed with diethyl ether (3 x 2 ml) and dried in vacuo. The targeted compound was isolated as a yellow solid.
Yield 88 mg (95%)

Version 5:
All procedures were performed under nitrogen atmosphere in pre-dried solvents.
Anhydrous uranyl acetate (55 mg, 142 µmol, 3.0 eq.) was added to a solution of H2L1 (41 mg, 144 µmol, 3.0 eq.) in methanol (5 ml). The reaction mixture was stirred at room temperature for 2 h, after which cesium acetate (20 mg, 104 µmol, 2.2 eq.) was added and the solution was stirred overnight. The yellow precipitate was filtered off, washed with diethyl ether (3 x 2 ml) and dried in vacuo. The product was isolated as a yellow solid.
Yield: 52 mg (57%)

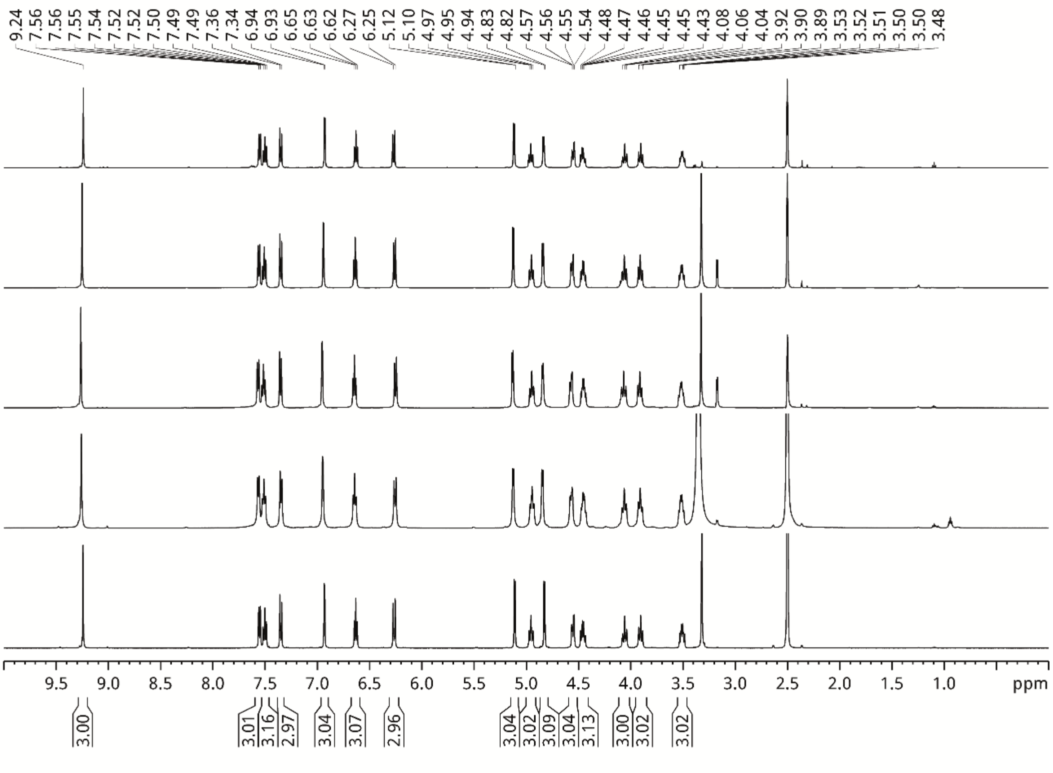

Figure S5: ^{1}H-NMR spectra of $[Cs]_2$**1** synthesized by the various methods; version 1 (bottom) to version 5 (top).

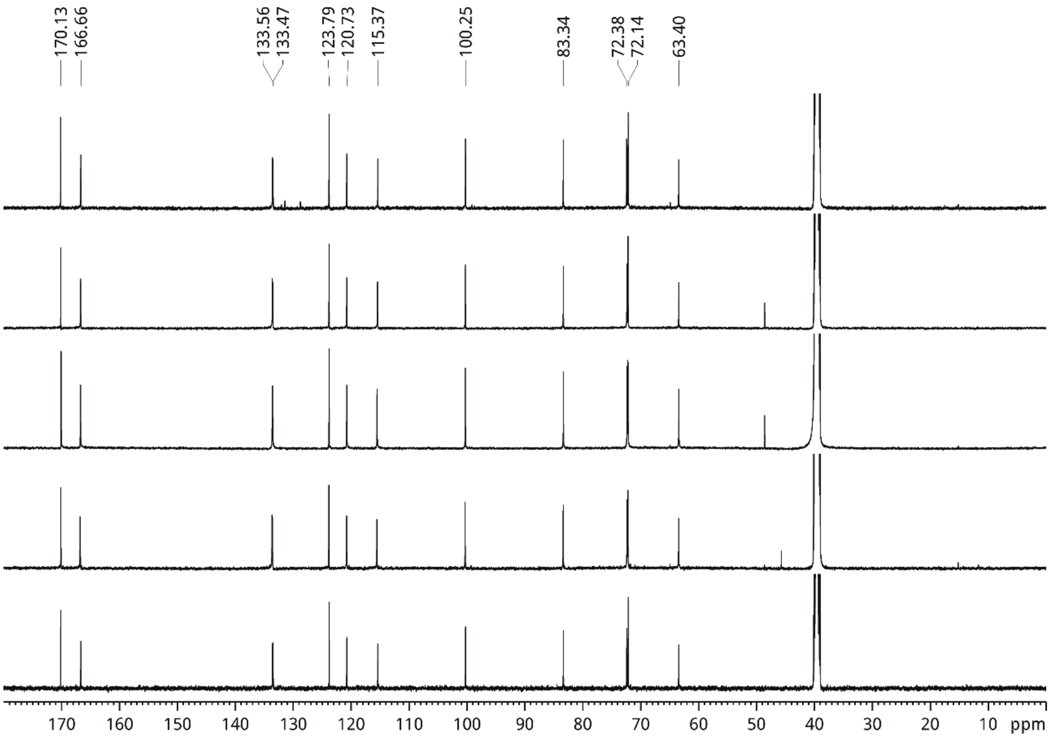

Figure S6: $^{13}C\{^{1}H\}$-NMR spectra of $[Cs]_2$**1** synthesized by the various methods; version 1 (bottom) to version 5 (top).

S2.5 Preparation of $[Cs(18\text{-crown-}6)]_2[(UO_2)_3(\mu_3\text{-}O)(L^1)_3]$ ($[Cs(18\text{-crown-}6)]_2$1)

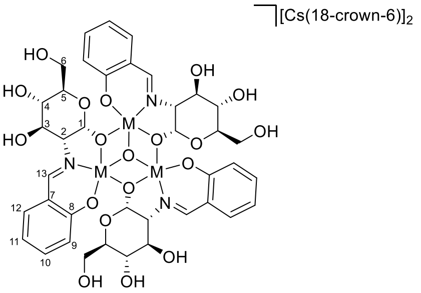

M = UO_2

H_2**L^1** (50 mg, 0.18 mmol, 4.5 eq.) was dissolved in methanol (3 ml). Uranyl acetate dihydrate (50 mg, 0.12 mmol, 3.0 eq.) was dissolved in methanol (4.5 ml) and slowly added to the reaction mixture. After stirring at room temperature for 2 h, a solution of cesium carbonate (27 mg, 0.08 mmol, 2.0 eq.) in methanol (1.5 ml) was added and the reaction mixture was stirred at room temperature for an additional 20 h. The yellow precipitate was then filtered off, washed with diethyl ether (3 x 2 ml) and dried in air. The dried solid was dissolved in dimethylformamide (1.0 ml) and 18-crown-6 (42 mg, 0.16 mmol, 4.0 eq.) was added. Crystals of 2($[Cs(18\text{-crown-}6)]_2$**1**•9DMF•$4H_2O$ were obtained by slow diffusion of diethyl ether vapor into the solution over 5 d. The crystalline solid was filtered off and washed with diethyl ether (5 x 2 ml) and dried in vacuo. The target compound was isolated as a crystalline yellow solid.

Yield: 74 mg (70%); **m.p.:** 275 °C (decomp.); **Raman** (100 mW, 1000 scans, 300 K, [cm^{-1}]) : 3052 (12), 3025 (6), 2887 (37), 2849 (19), 1628 (100), 1599 (28), 1549 (55), 1474 (36), 1451 (29), 1396 (9), 1356 (8), 1327 (53), 1300 (12), 1275 (13), 1254 (15), 1208 (13), 1148 (14), 1120 (9), 1084 (8), 1032 (31), 970 (13), 940 (8), 867 (11), 817 (60), 806 (54), 784 (16), 762 (6), 596 (8), 571 (8), 553 (10), 517 (11), 478 (11), 416 (12), 340 (11), 295 (13), 242 (16), 206 (23), 159 (20) **IR** (ATR, 300 K, [cm^{-1}]): 3375 (vw), 2886 (w), 2846 (w), 1625 (m), 1597 (w), 1547 (w), 1471 (w), 1448 (w), 1420 (w), 1396 (w), 1351 (w), 1322 (w), 1299 (m), 1289 (m), 1244 (w), 1199 (vw), 1134 (m), 1104 (vs), 1090 (s), 1026 (s), 993 (m), 958 (m), 937 (w), 894 (vs), 862 (m), 838 (s), 802 (s), 758 (m), 741 (w), 686 (w), 655 (w), 593 (m), 570 (w), 552 (m), 514 (vs), 501 (vs), 444 (s), 432 (s), 416 (m); **^{1}H-NMR** (500 MHz, DMSO-d_6, 300 K, in ppm): δ = 2.70-2.97 (48H, m(br.), $v_{1/2}\approx$ 43 Hz, 18-crown-6), 3.54 (3H, *pseudo*-dt, $^3J_{3H,4H}$ = 8.7 Hz, $^3J_{4H,5H}$ = 9.5 Hz, $^3J_{4H,4OH}$ = 3.6 Hz, 4 H), 3.88 (3H, *pseudo*-t, $^2J_{6H,6H'}$ = 9.1 Hz, $^3J_{5H,6H}$ = 10.0 Hz, 6 H), 3.97 (3H, *pseudo*-t, $^2J_{6H,6H'}$ = 9.1 Hz, $^3J_{6H',6OH}$ = 10.1 Hz, 6 H'), 4.35-4.50 (3H, m, 3 H), 4.59 (3H, dd, $^3J_{2H,3H}$ = 9.1 Hz, $^3J_{1H,2H}$ = 3.4 Hz, 2 H), 4.93 (3H, d, $^3J_{4H,4OH}$ = 3.6 Hz, 4 OH), 5.02 (3H, *pseudo*-t, $^3J_{5H,6H}$ = 10.0 Hz, $^3J_{4H,5H}$ = 9.5 Hz, 5 H), 5.08 (3H, d, $^3J_{3H,3OH}$ = 5.3 Hz, 3 OH), 6.26 (3H, d, $^3J_{6H',6OH}$ = 10.1 Hz, 6 OH), 6.61 (3H, *pseudo*-t, $^3J_{11H,12H}$ = 7.7 Hz, $^3J_{10H,11H}$ = 7.0 Hz, 11 H), 6.89 (3H, d, $^3J_{1H,2H}$ = 3.4 Hz, 1 H), 7.35 (3H, d, $^3J_{9H,10H}$ = 8.4 Hz, 9 H), 7.48 (3H, ddd, $^3J_{9H,10H}$ = 8.4 Hz, $^3J_{10H,11H}$ = 7.0 Hz, $^4J_{10H,12H}$ = 1.5 Hz, 10 H), 7.54 (3H, dd, $^3J_{11H,12H}$ = 7.7 Hz, $^4J_{10H,12H}$ = 1.5 Hz, 12 H), 9.25 (3H, s, 13 H); **$^{13}C\{^1H\}$-NMR** (125 MHz, DMSO-d_6, 300 K, in ppm): δ = 63.8 (3C, s, 6 C), 68.9 (24C, s, 18-crown-6), 71.7 (3C, s, 5 C), 72.5 (3C, s, 4 C), 72.6 (3C, s, 3 C), 83.5 (3C, s, 2 C), 100.3 (3C, s, 1-C), 115.7 (3C, s, 11 C), 120.4 (3C, s, 9 C), 123.8 (3C, s, 7 C), 133.5 (6C, s, 10 C, 12 C), 166.7 (3C, s, 13 C), 170.0 (3C, s, 8 C); **ESI-MS:** m/z [Da/e] = 834.7 (**1**$^{2-}$), 1672.2 ({**1**+H}$^-$), 1802.2 ({**1**+Cs}); **Elemental Analysis:** calculated for $[Cs(18\text{-crown-}6)]_2$**1** ($C_{63}H_{93}Cs_2N_3O_{37}U_3$) C: 30.71%, H: 3.80%, N: 1.71%, found C: 30.62%, H: 3.82%, N: 1.94%.

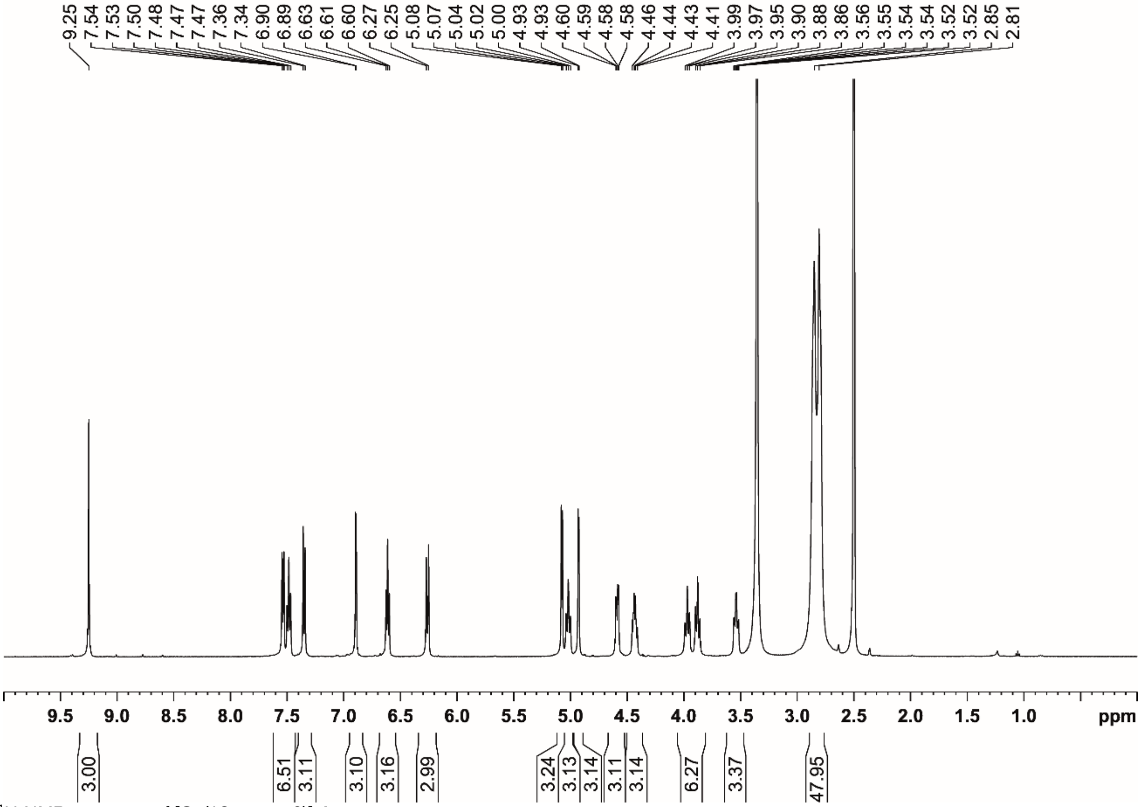

Figure S7: ^{1}H-NMR spectrum of [Cs(18-crown-6)]$_2$**1**.

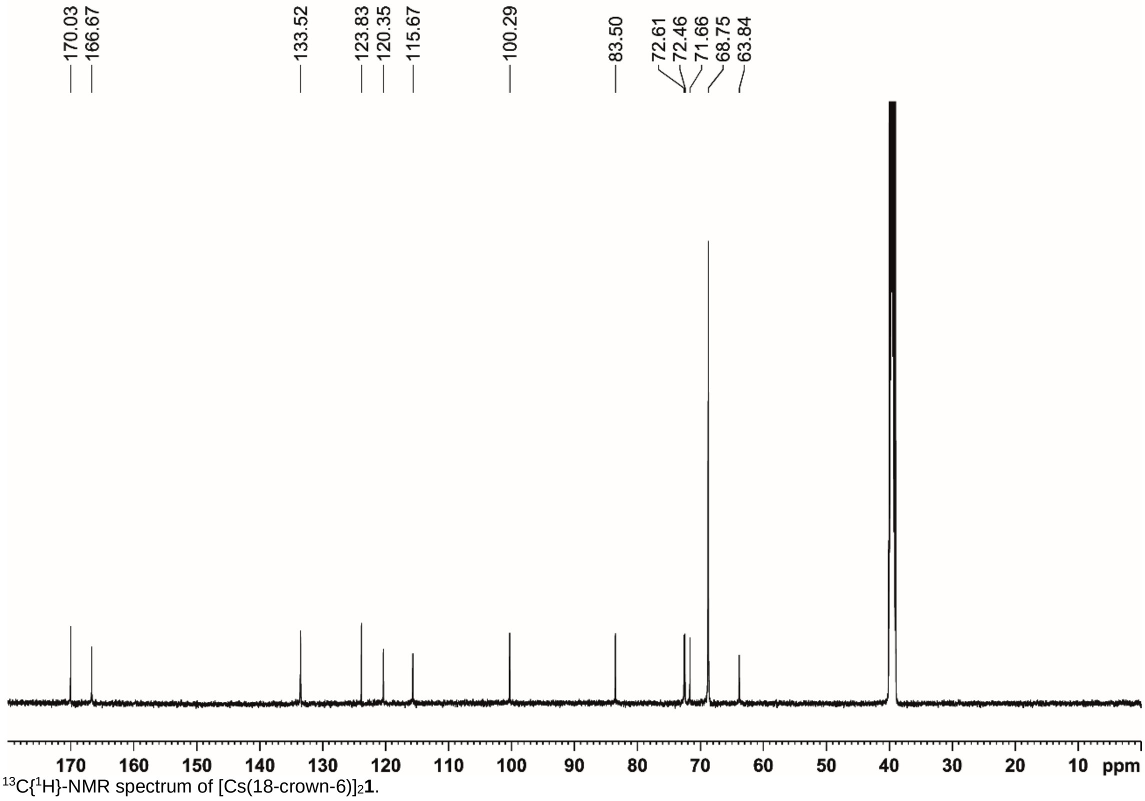

Figure S8: ^{13}C{^{1}H}-NMR spectrum of [Cs(18-crown-6)]$_2$**1**.

S2.6 Preparation of $[Rb(18\text{-crown-}6)]_2[(UO_2)_3(\mu_3\text{-}O)(L^1)_3]\cdot0.5DMF$ ($[Rb(18\text{-crown-}6)]_2$**1**$\cdot$0.5DMF)

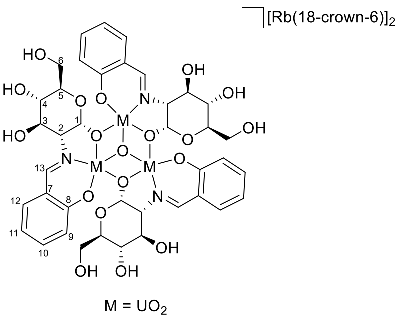

H_2**L**^{1} (50 mg, 0.18 mmol, 4.5 eq.) was dissolved in methanol (3 ml). Uranyl acetate dihydrate (50 mg, 0.12 mmol, 3.0 eq.) dissolved in methanol (4.5 ml) was slowly added and the reaction mixture was stirred at room temperature for 2 h. Rubidium acetate (24 mg, 0.16 mmol, 4.0 eq.) was dissolved in methanol (1.5 ml) and added to the reaction mixture. After stirring at room temperature for 20 h, additional solid rubidium acetate (38 mg, 0.26 mmol, 6.6 eq.) was added and the reaction mixture stirred at room temperature for an additional 22 h. The yellow precipitate was filtered off, washed with diethyl ether (3 x 2 ml) and dried in air. The dried solid was dissolved in dimethylformamide (1.0 ml) and 18 crown 6 (42 mg, 0.16 mmol, 4.0 eq.) was added. Crystals of $[Rb(18\text{-crown-}6)]_2$**1**•3DMF•$2H_2O$ were obtained by slow diffusion of diethyl ether vapour into the solution over two months. The crystalline solid was filtered off and washed with diethyl ether (5 x 2 ml) and dried in vacuo. $[Rb(18\text{-crown-}6)]_2$**1**•0.5 DMF was obtained as crystalline yellow solid (33 mg, 14 µmol, yield: 35%).

Yield: 33 mg (35%); **m.p.:** 236 °C (decomp.); **Raman** (100 mW, 1000 scans, 300 K, [cm^{-1}]) : 3047 (19), 2892 (49), 2850 (40), 2811 (21), 1626 (100), 1596 (42), 1549 (77), 1473 (63), 1449 (39), 1412 (18), 1327 (58), 1274 (25), 1251 (26), 1202 (25), 1149 (23), 1086 (18), 1031 (40), 970 (16), 942 (16), 867 (21), 816 (63), 803 (75), 782 (21), 596 (16), 552 (18), 517 (16), 479 (19), 432 (16), 416 (19), 342 (18), 299 (21), 206 (30); **IR** (ATR, 300 K, [cm^{-1}]): 3302 (w), 2853 (w), 1661 (w), 1622 (m), 1595 (w), 1547 (w), 1471 (w), 1447 (w), 1351 (m), 1301 (m), 1251 (w), 1200 (w), 1135 (m), 1103 (vs), 1026 (s), 992 (m), 958 (m), 892 (vs), 838 (s), 801 (s), 759 (m), 742 (m), 684 (w), 656 (w), 593 (w), 570 (w), 552 (m), 513 (vs); **^{1}H-NMR** (500 MHz, DMSO-d_6, 300 K, in ppm): δ = 2.10-3.40 (54H, m(br.), $\nu_{1/2}\approx$ 250 Hz, 18-crown-6, DMF-N(CH_3)$_2$), 3.55 (3H, *pseudo*-dt, $^3J_{3H,4H}$ = 9.0 Hz, $^3J_{4H,5H}$ = 9.3 Hz, $^3J_{4H,4OH}$ = 3.8 Hz, 4-H), 3.90 (3H, *pseudo*-t, $^2J_{6H,6H'}$ = 9.5 Hz, $^3J_{5H,6H}$ = 9.4 Hz, 6 H), 3.97 (3H, *pseudo*-t, $^2J_{6H,6H'}$ = 9.5 Hz, $^3J_{6H',6OH}$ = 10.1 Hz, 6 H'), 4.45-4.49 (3H, m, 3-H), 4.57 (3H, dd, $^3J_{1H,2H}$ = 3.3 Hz, $^3J_{2H,3H}$ = 9.1 Hz, 2-H), 5.00 (3H, d, $^3J_{4H,4OH}$ = 3.8 Hz, 4-OH), 5.10 (3H, *pseudo*-t, $^3J_{4H,5H}$ = 9.4 Hz, $^3J_{5H,6H}$ = 9.3 Hz, 5-H), 5.14 (3H, d, $^3J_{3H,3OH}$ = 5.2 Hz, 3-OH), 6.35 (3H, d, $^3J_{3H,4H}$ = 10.1 Hz, 6-OH), 6.63 (3H, *pseudo*-t, ^3J10H,11H = 7.0 Hz, $^3J_{11H,12H}$ = 7.5 Hz, 11-OH), 6.89 (3H, d, $^3J_{1H,2H}$ = 3.3 Hz, 1-H), 7.39 (3H, d, $^3J_{9H,10H}$ = 8.4 Hz, 9-H), 7.48, (3H, *pseudo*-dt, $^3J_{9H,10H}$ = 8.4 Hz, $^3J_{10H,11H}$ = 7.0 Hz, $^4J_{10H,12H}$ = 1.2 Hz, 10-H), 7.55 (3H, dd, $^3J_{11H,12H}$ = 7.5 Hz, $^4J_{10H,12H}$ = 1.2 Hz, 12-H), 7.95 (1H, s, DMF), 9.25 (3H, s, 13-H); **^{13}C{^{1}H}-NMR** (125 MHz, DMSO-d_6, 300 K, in ppm): δ = 30.7 (1C, s, DMF-N(CH_3)), 35.7 (1C, s, DMF-N(CH_3)), 63.7 (3C, s, 6-C), 68.7 (24C, s, 18-crown-6), 71.9 (3C, s, 5-C), 72.3 (3C, s, 4-C), 72.4 (3C, s, 3-C), 83.5 (3C, s, 2-C), 100.3 (3C, s, 1-C), 115.8 (3C, s, 11-C), 120.4 (3C, s, 9-C), 123.9 (3C, s, 7-C), 133.5 (3C, s, 10-C), 133.6 (3C, s, 12-C), 162.3 (1C, s, DMF-CO), 166.8 (3C, s, 13-C), 169.8 (3C, s, 8-C), 185.3; **ESI-MS:**:m/z [Da/e] = 834.9 ($[(UO_2)_3O(\mathbf{L}^1)_3]^{2-}$), 1670.6 ($\{[(UO_2)_3O(\mathbf{L}^1)_3]+H\}^-$), 1724.6 ($\{[(UO_2)_3O(\mathbf{L}^1)_3]+Rb\}^-$); **Elemental Analysis:** calculated for $[Rb(18\text{-crown-}6)]_2$**1**•0.5DMF ($C_{63}H_{93}Rb_2N_3O_{37}U_3$ x 0.5 C_3H_7NO) calculated C: 32.2 71%, N: 2.04%, H: 4.04%, found C: 31.96%, N: 1.86%, H: 4.00%

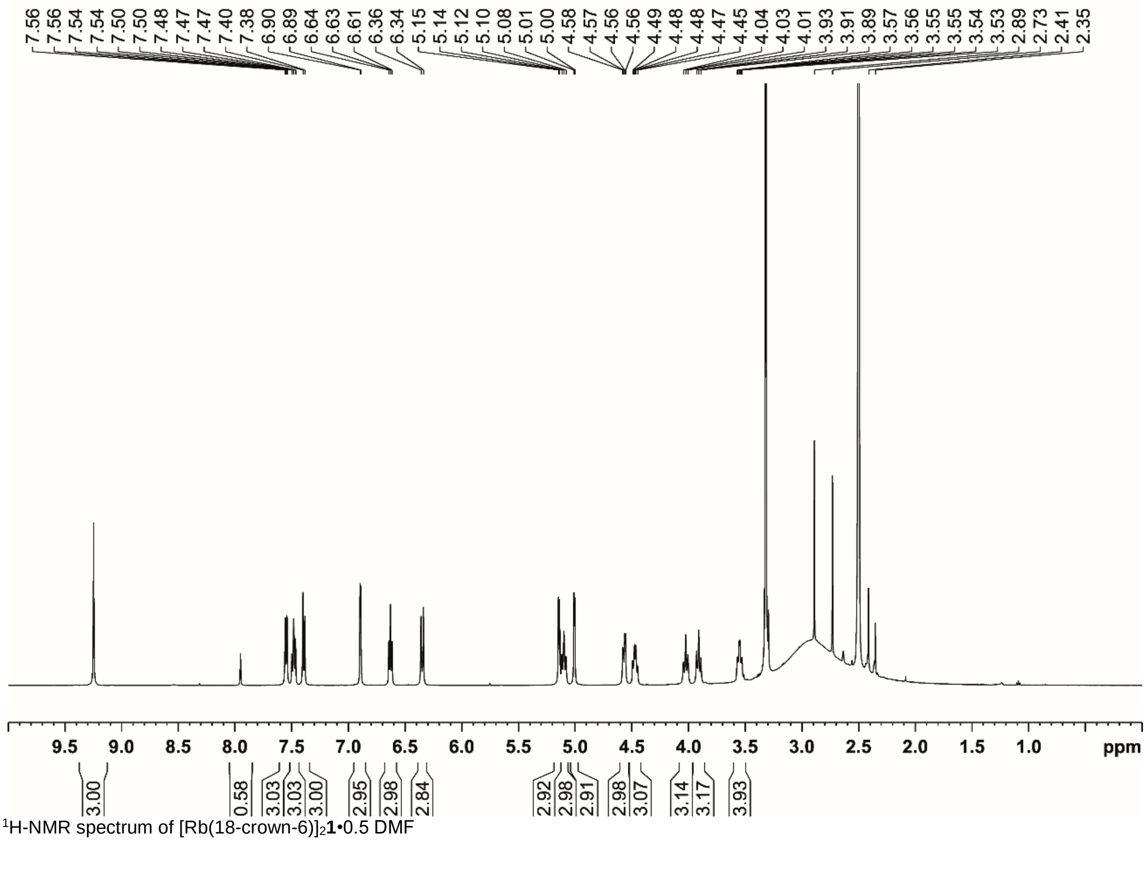

Figure S9: ^{1}H-NMR spectrum of $[Rb(18\text{-crown-}6)]_2$**1**•0.5 DMF

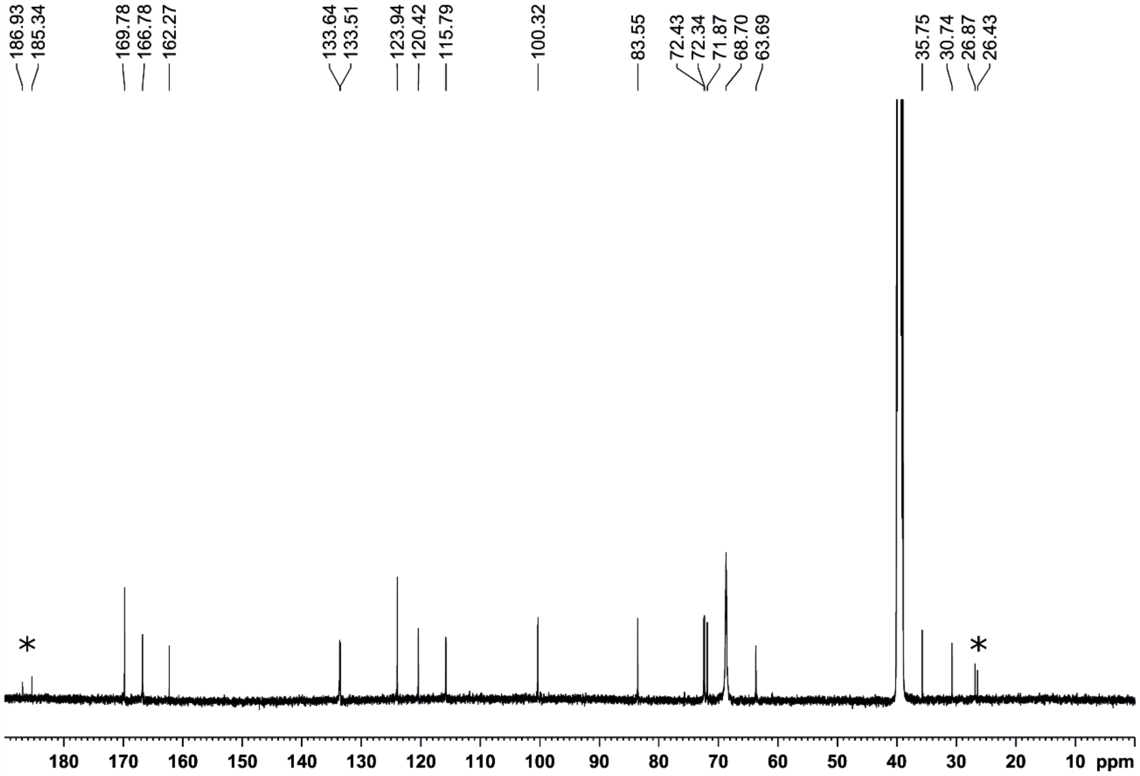

Figure S10: $^{13}C\{^{1}H\}$-NMR spectrum of $[Rb(18\text{-crown-}6)]_2$**1**•0.5 DMF

S2.7 Preparation of $[K(18\text{-crown-}6)]_2[(UO_2)_3(\mu_3\text{-}O)(L^1)_3]$ ($[K(18\text{-crown-}6)]_2$1)

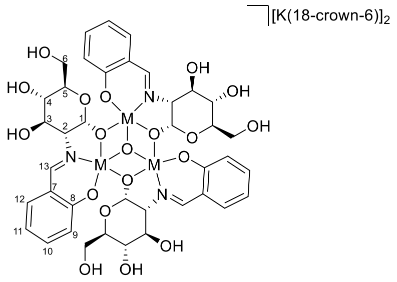

M = UO_2

H_2**L¹** (56 mg, 0.20 mmol, 4.5 eq.) was dissolved in methanol (3 ml). Uranyl acetate dihydrate (55 mg, 0.13 mmol, 3.0 eq.) dissolved in methanol (4.5 ml) was slowly added to the above solution and the mixture was stirred at room temperature for 2 h. Potassium acetate (20 mg, 0.20 mmol, 4.5 eq.) was dissolved in methanol (1.5 ml) and added to the reaction mixture. After stirring the solution at room temperature for 20 h, the yellow precipitate was filtered off, washed with diethyl ether (3 x 2 ml) and dried in air. The dried solid was dissolved in dimethylformamide (2.5 ml) and 18-crown-6 (42 mg, 0.16 mmol, 4.0 eq.) was added. The target compound was crystallized by slow diffusion of diethyl ether vapor into the solution over 5 d. The crystalline solid was filtered off and washed with diethyl ether (5 x 2 ml) then dried in vacuo. The target compound was isolated as a crystalline yellow solid.

Yield: 49 mg (50%); **m.p.:** 252 °C (decomp.); **Raman** (100 mW, 1000 scans, 300 K, [cm^{-1}]): 3105 (8), 3049 (21), 3028 (12), 2895 (54), 2856 (31), 2816 (17), 1628 (100), 1597 (42), 1576 (19), 1550 (81), 1473 (56), 1451 (40), 1399 (15), 1362 (15), 1327 (67), 1273 (15), 1252 (23), 1205 (19), 1151 (19), 1086 (12), 1031 (35), 971 (12), 944 (12), 869 (12), 804 (44), 785 (33), 597 (12), 572 (10), 552 (12), 516 (12), 479 (13), 436 (12), 416 (12), 341 (12), 300 (15), 246 (13), 212 (17), 187 (15), 167 (15), 121 (35); **IR** (ATR, 300 K, [cm^{-1}]): 3309 (vw), 2858 (vw), 1624 (w), 1596 (w), 1548 (vw), 1471 (vw), 1448 (w), 1399 (vw), 1353 (w), 1325 (vw), 1296 (w), 1247 (w), 1136 (w), 1105 (m), 1096 (m), 1032 (m), 995 (w), 961 (w), 941 (w), 886 (s), 864 (w), 846 (w), 800 (m), 786 (w), 778 (w), 759 (m), 743 (w), 686 (w), 657 (w), 595 (w), 573 (w), 551 (w), 515 (vs), 500 (vs), 459 (m), 443 (s), 418 (m); **^{1}H-NMR** (500 MHz, DMSO-d_6, 300 K, in ppm): δ = 2.73 (48H, s(br.), $\nu_{1/2}\approx$ 190 Hz, 18-crown-6), 3.52 (3H, *pseudo*-dt, $^3J_{3H,4H}$ = 8.6 Hz, $^3J_{4H,5H}$ = 9.1 Hz, $^3J_{4H,4OH}$ = 5.2 Hz, 4-H), 3.91 (3H, *pseudo*-t, $^3J_{5H,6H}$ = 9.6 Hz, $^2J_{6H,6H'}$ = 9.3 Hz, 6-H), 4.08 (3H, *pseudo*-t, $^3J_{6H',6OH}$ = 9.9 Hz, $^2J_{6H,6H'}$ = 9.3 Hz, 6-H'), 4.49 (3H, dd, $^3J_{1H,2H}$ = 3.0 Hz, $^3J_{2H,3H}$ = 9.1 Hz, 2-H), 4.51-4.60 (3H, m, 3-H), 4.99 (3H, d, $^3J_{4H,4OH}$ = 5.2 Hz, 4-OH), 5.12 (3H, dd, $^3J_{4H,5H}$ = 9.1 Hz, $^3J_{5H,6H}$ = 9.6 Hz, 5-H), 5.18 (3H, d, $^3J_{3H,3OH}$ = 5.1 Hz, 3-OH), 6.34 (3H, d, $^3J_{6H',6OH}$ = 9.9 Hz, 6-OH), 6.61 (3H, *pseudo*-t, $^3J_{10H,11H}$ = 7.2 Hz, $^3J_{11H,12H}$ = 7.2 Hz, 11-H), 6.82 (3H, d, $^3J_{1H,2H}$ = 3.0 Hz, 1-H), 7.39 (3H, d, $^3J_{9H,10H}$ = 8.4 Hz, 9 H), 7.45 (3H, *pseudo*-t, $^3J_{9H,10H}$ = 8.4 Hz, $^3J_{10H,11H}$ = 7.2 Hz, $^4J_{10H,12H}$ = 1.2 Hz, 10 H), 7.51 (3H, dd, $^3J_{11H,12H}$ = 7.2 Hz, $^4J_{10H,12H}$ = 1.2 Hz, 12 H), 9.20 (3H, s, 13 H); **$^{13}C\{^{1}H\}$-NMR** (500 MHz, DMSO-d_6, 300 K, in ppm): δ = 63.5 (3C, s, 6-C), 68.4 (24C, s(br.), $\nu_{1/2}\approx$ 27 Hz, 18-crown-6), 72.0 (3C, s, 5 C), 72.1 (3C, s, 3 C), 72.5 (3C, s, 4 C), 83.6 (3C, s, 2 C), 100.2 (3H, s, 1 C), 115.4 (3C, s, 11 C), 120.8 (3C, s, 9 C), 124.0 (3C, s, 7 C), 133.4 (3C, s, 10 C), 133.7 (3C, s, 12 C), 166.8 (3C, s, 13 C), 170.0 (3C, s, 8 C); **Elemental Analysis:** calculated for $[K(18\text{-crown-}6)]_2$**1** ($C_{63}H_{93}N_3O_{37}U_3K_2$) C: 33.24%, N: 1.85%, H: 4.12%, found: C: 31.05%, N: 1.95%, H: 3.79%

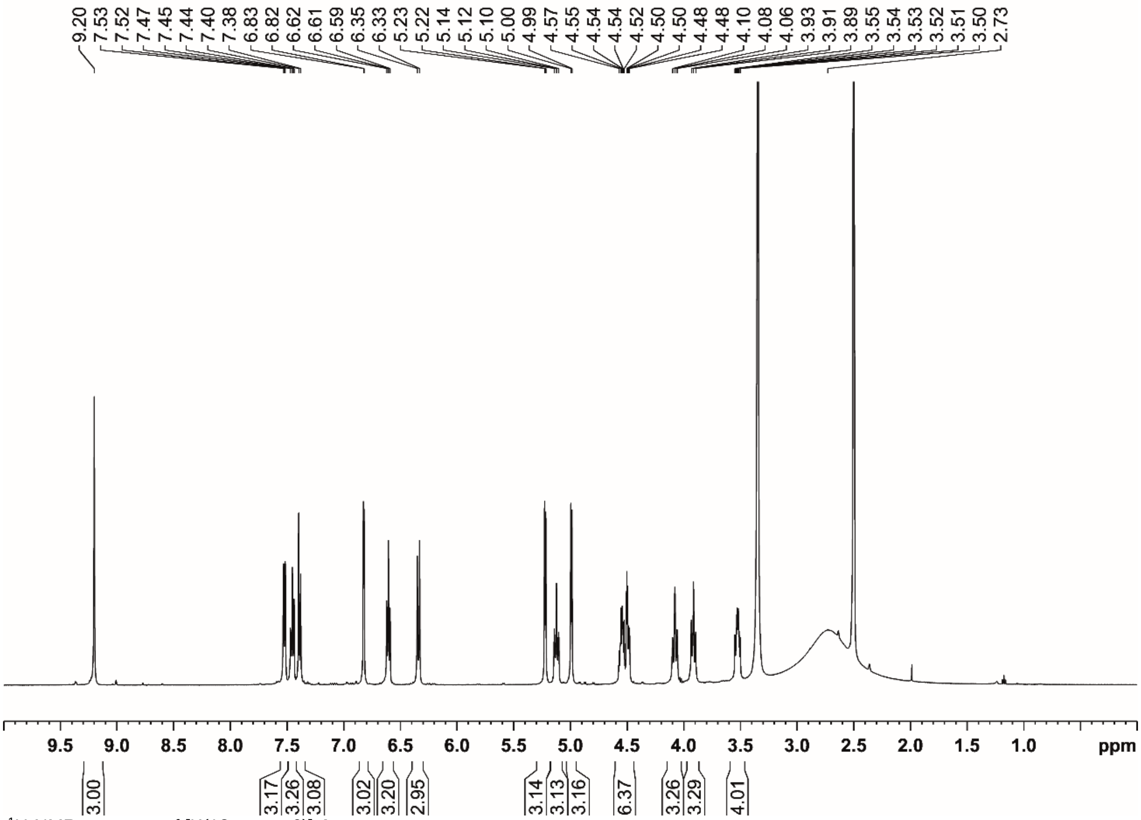

Figure S11: ^{1}H-NMR spectrum of [K(18-crown-6)]$_2$**1**.

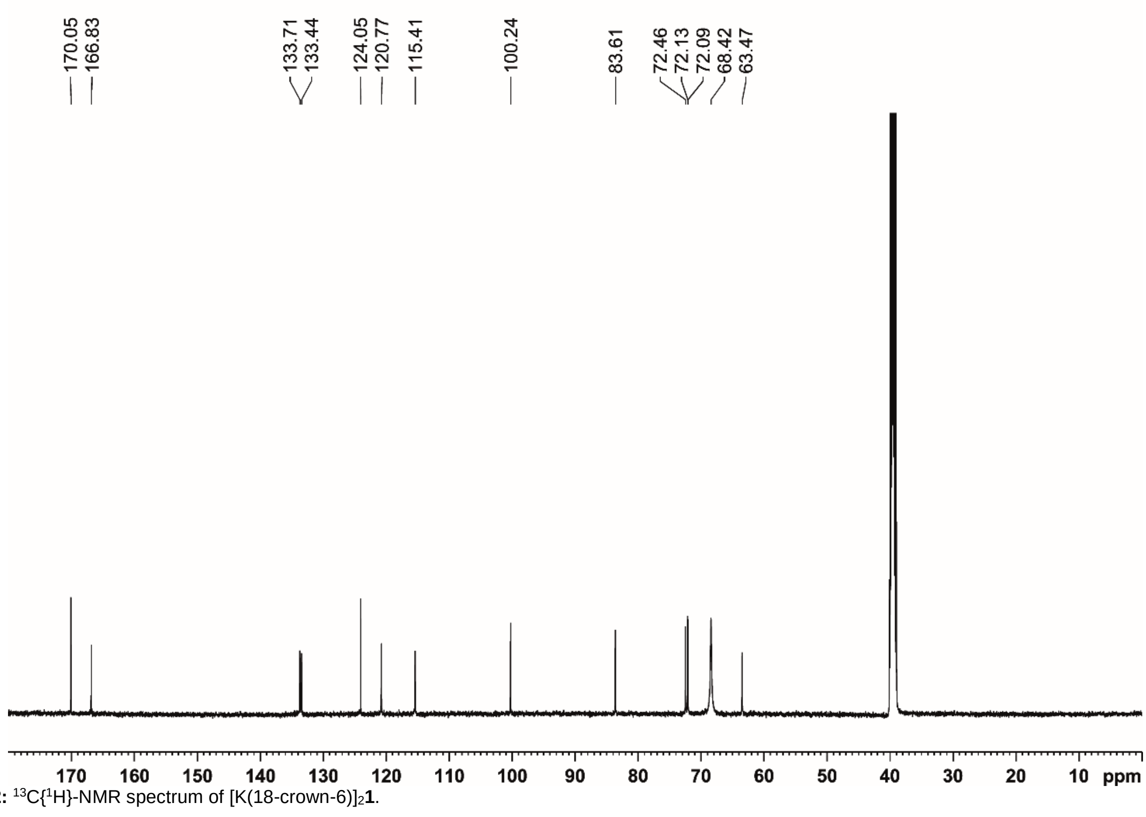

Figure S12: ^{13}C{^{1}H}-NMR spectrum of [K(18-crown-6)]$_2$**1**.

S2.8 Preparation of $[Cs(18\text{-crown-}6)]_2[(UO_2)_3(\mu_3\text{-}O)(L^2)_3]$ ($[Cs(18\text{-crown-}6)]_2$**2**)

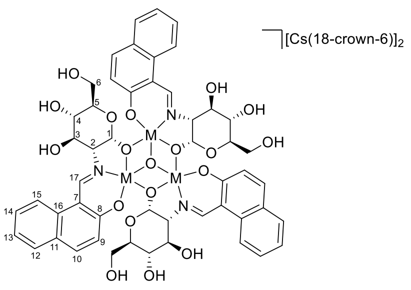

M = UO_2

H_2**L**^{2} (62 mg, 0.18 mmol, 4.5 eq.) was dissolved in methanol (10 ml). Uranyl acetate dihydrate (52 mg, 0.12 mmol, 3.0 eq.) was dissolved in methanol (4.5 ml) and slowly added to the above solution. After stirring the mixture at room temperature for 2 h, a solution of cesium carbonate (33 mg, 0.10 mmol, 2.5 eq.) in methanol (1.5 ml) was added and the reaction mixture was stirred for an additional 22 h. The orange precipitate was then filtered off, washed with diethyl ether (3 x 2 ml) and dried in air. The dried solid was dissolved in in dimethylformamide (5 ml) and 18-crown-6 (46 mg, 0.18 mmol, 4.5 eq.) was added. Crystals of $[Cs(18\text{-crown-}6)]_2$**2**•DMF•$H_2O$ were obtained by slow diffusion of diethyl ether vapor into the solution over 5 d. The crystalline solid was filtered off and washed with diethyl ether (5 x 2 ml) then dried in vacuo. The target compound was isolated as a crystalline yellow solid.

Yield: 67 mg (64%); **m.p.:** 241 °C (decomp.); **Raman** (100 mW, 1000 scans, 300 K, [cm^{-1}]): 3066 (11), 2877 (21), 1613 (48), 1548 (61), 1472 (15), 1456 (22), 1435 (62), 1386 (100), 1362 (40), 1274 (9), 1249 (8), 1143 (11), 1038 (11), 944 (14), 865 (10), 816 (44), 768 (17), 688 (8), 653 (9), 544 (13), 508 (19), 425 (12), 281 (13) **IR** (ATR, 300 K, [cm^{-1}]): 3344 (w), 2873 (w), 1620 (m), 1547 (w), 1455 (w), 1432 (w), 1417 (w), 1385 (w), 1350 (m), 1338 (m), 1297 (m), 1248 (w), 1107 (vs), 1029 (vs), 991 (w), 979 (m), 957 (m), 896 (vs), 834 (s), 745 (m), 511 (vs), 461 (s), 446 (vs), 397 (s); **^{1}H-NMR** (500 MHz, DMSO-d_6, 300 K, in ppm): δ = 2.40-2.80 (48H, s(br.), $\nu_{1/2}\approx$ 55 Hz, 18-crown-6), 3.63 (3H, *pseudo*-dt, $^3J_{3H,4H}$ = 8.5 Hz, $^3J_{4H,4OH}$ = 3.5 Hz, $^3J_{4H,5H}$ = 9.6 Hz, 4 H), 3.98 (3H, *pseudo*-t, $^3J_{5H,6H}$ = 10.0 Hz, $^3J_{6H,6H'}$ = 8.7 Hz, 6-H), 4.06 (3H, *pseudo*-t, $^3J_{6H,6H'}$ = 8.7 Hz, $^3J_{6H',6OH}$ = 9.9 Hz, 6-H'), 4.56 (3H, *pseudo*-q, $^3J_{2H,3H}$ = 9.1 Hz, $^3J_{3H,3OH}$ = 5.1 Hz, $^3J_{3H,4H}$ = 8.5 Hz, 3-H), 4.92 (3H, dd, $^3J_{1H,2H}$ = 3.0 Hz, $^3J_{2H,3H}$ = 9.1 Hz, 2-H), 4.98, (3H, d, $^3J_{4H,4OH}$ = 3.5 Hz, 4-OH), 5.05-5.15 (6H, m, 3-OH, 5-H), 6.07 (3H, d, $^3J_{6H',6OH}$ = 9.9 Hz, 6-OH), 6.96 (3H, d, $^3J_{1H,2H}$ = 3.0 Hz, 1-H), 7.16 (3H, *pseudo*-t, $^3J_{13H,14H}$ = 7.4 Hz, $^3J_{14H,15H}$ = 7.4 Hz, 14-H), 7.43 (3H, *pseudo*-t, $^3J_{12H,13H}$ = 8.5 Hz, $^3J_{13H,14H}$ = 7.4 Hz, 13-H), 7.76 (6H, *pseudo*-t, $^3J_{9H,10H}$ = 9.2 Hz, $^3J_{9H,10H}$ = 9.2 Hz, $^3J_{14H,15H}$ = 7.4 Hz, 10-H, 15-H), 8.04 (3H, d, $^3J_{9H,10H}$ = 9.2 Hz, 9-H), 8.27 (3H, d, $^3J_{12H,13H}$ = 8.5 Hz, 12-H), 10.15 (3H, s, 17-H) **^{13}C{^{1}H}-NMR** (125 MHz, DMSO-d_6, 300 K, in ppm): δ = 63.77 (3C, 6-C), 68.53 (24C, 18-crown-6), 71.85 (3C, 5-C), 72.48 (3C, 4-C), 72.74 (3C, 3-C), 83.32 (3C, 2-C), 100.79 (3C, 1-C), 112.85 (3C, 16-C), 120.42 (3C, 12-C), 121.59 (3C, 14-C), 124.32 (3C, 15-C), 126.65 (3C, 13-C), 126.90 (3C, 7-C), 128.37 (3C, 10-C), 133.79 (3C, 9-C), 134.32 (3C, 11-C), 161.73 (3C, 17-C), 170.59 (3C, 8-C), **ESI-MS:** m/z [Da/e] = 910.0 ($[(UO_2)_3O(\mathbf{L}^2)_3]^{2-}$), 1820.8 ($\{[(UO_2)_3O(\mathbf{L}^2)_3]+H\}^-$); **Elemental Analysis:** calculated for $[Cs(18\text{-crown-}6)]_2$**2** ($C_{75}H_{99}N_3O_{37}U_3Cs_2$) C: 34.45%, H: 3.82%, N: 1.61%, found C: 34.05%, H: 3.93%, N: 1.60%

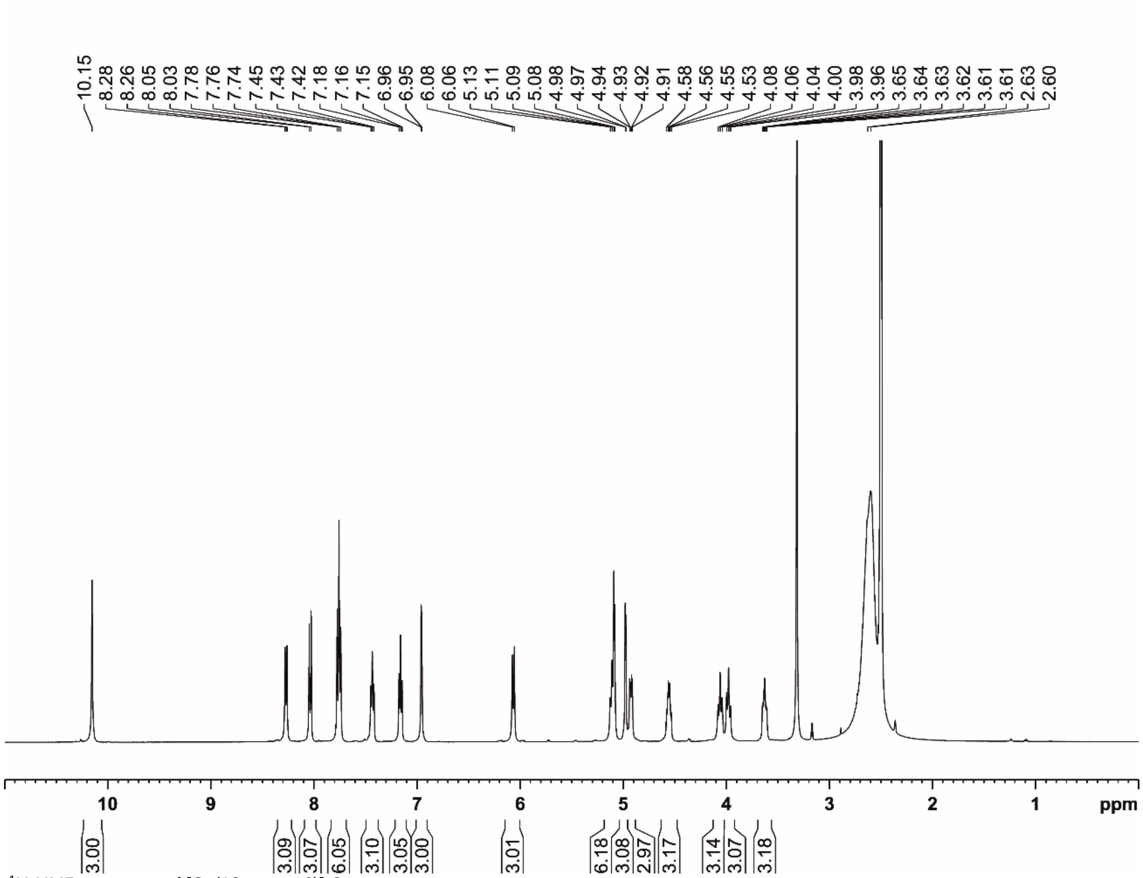

Figure S13: ^{1}H-NMR spectrum of $[Cs(18\text{-crown-}6)]_2$**2**.

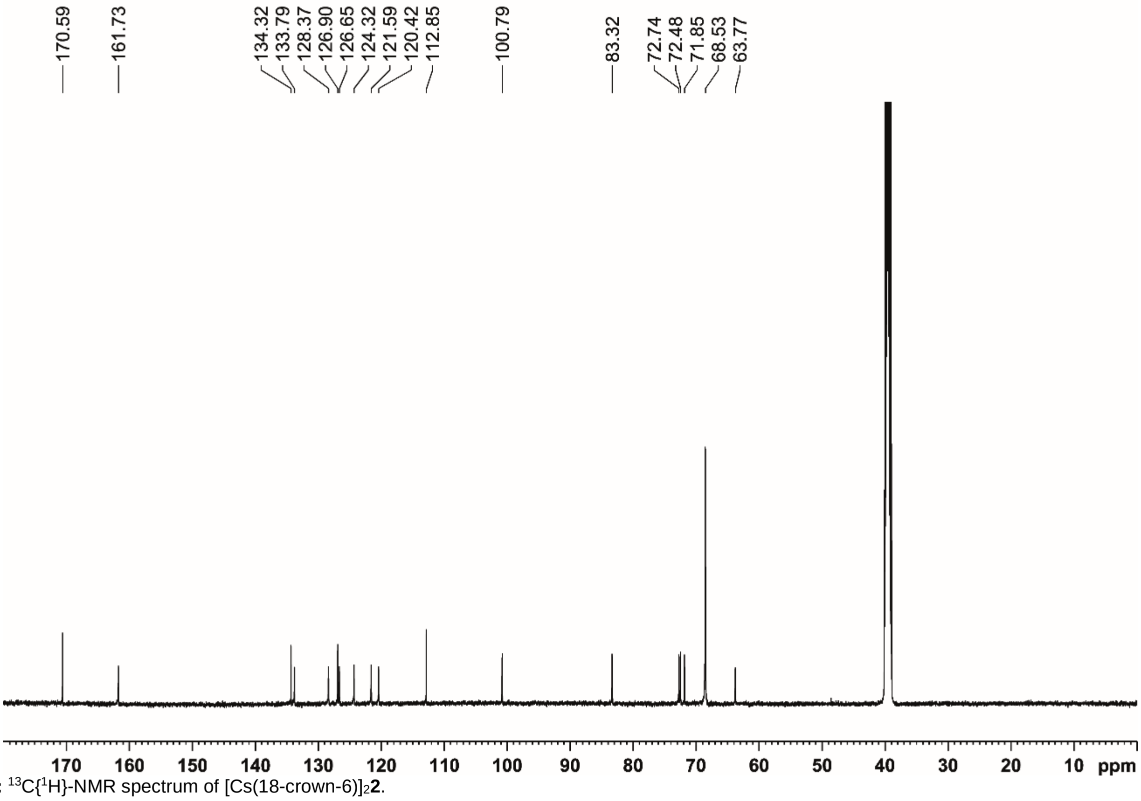

Figure S14: $^{13}C\{^{1}H\}$-NMR spectrum of $[Cs(18\text{-crown-}6)]_2$**2**.

S3. Additional spectroscopic data

S3.1 Job plot experiments of UO_2^{2+} and H_2L^2

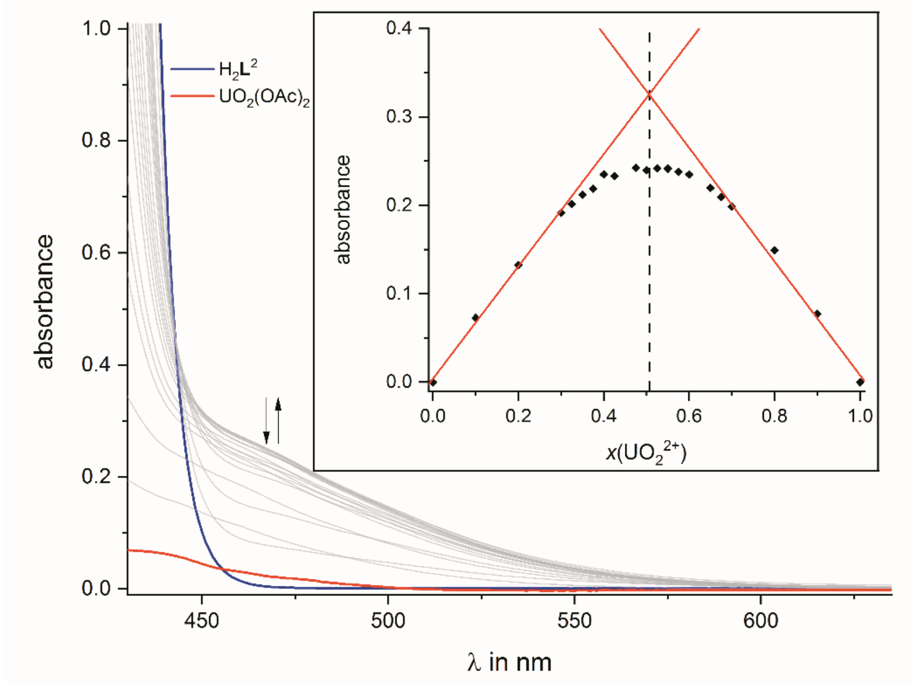

Figure S15: UV/Vis-Job plot for the interaction of H_2**L**^{2} and uranyl acetate $(UO_2(OAc)_2)\cdot 2H_2O$ in MeOH; the absorbance at 465 nm is plotted as a function of the UO_2^{2+} molar ratio.

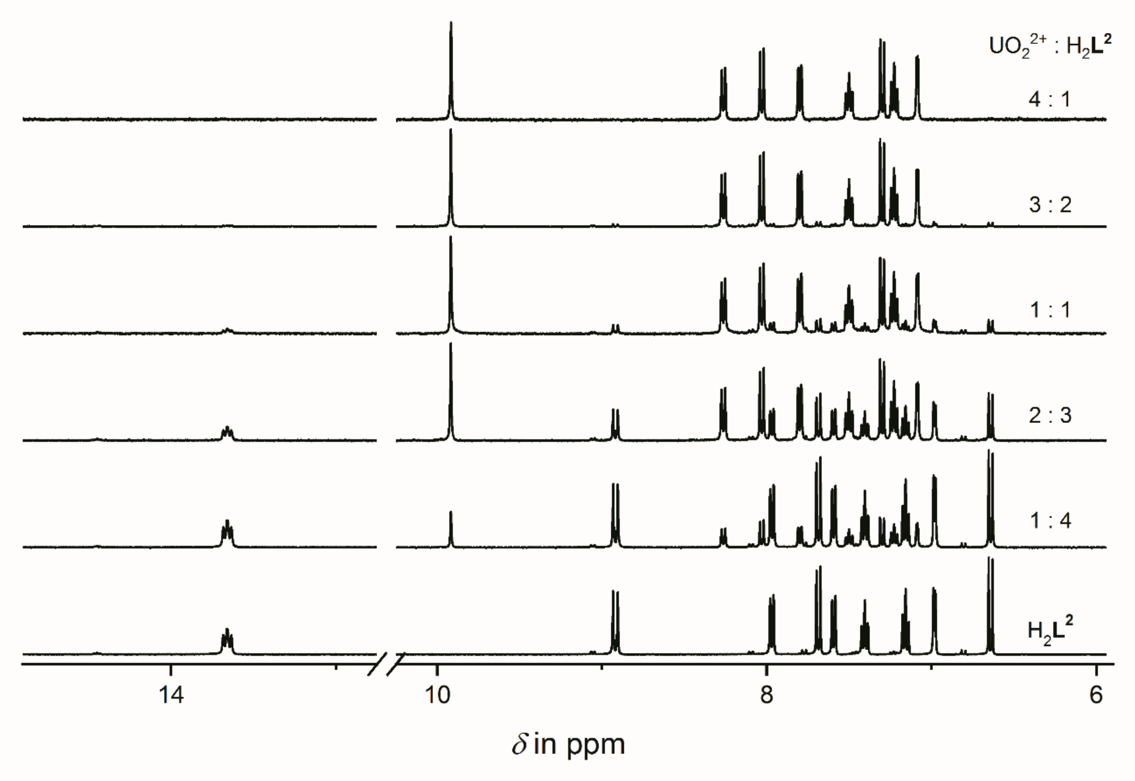

Figure S16: Excerpts of the ^{1}H-NMR spectra obtained during the investigation of complex formation between H_2**L^2** and $UO_2(OAc)_2$•$2H_2O$ in DMSO-d_6, employing the method of continuous variation.

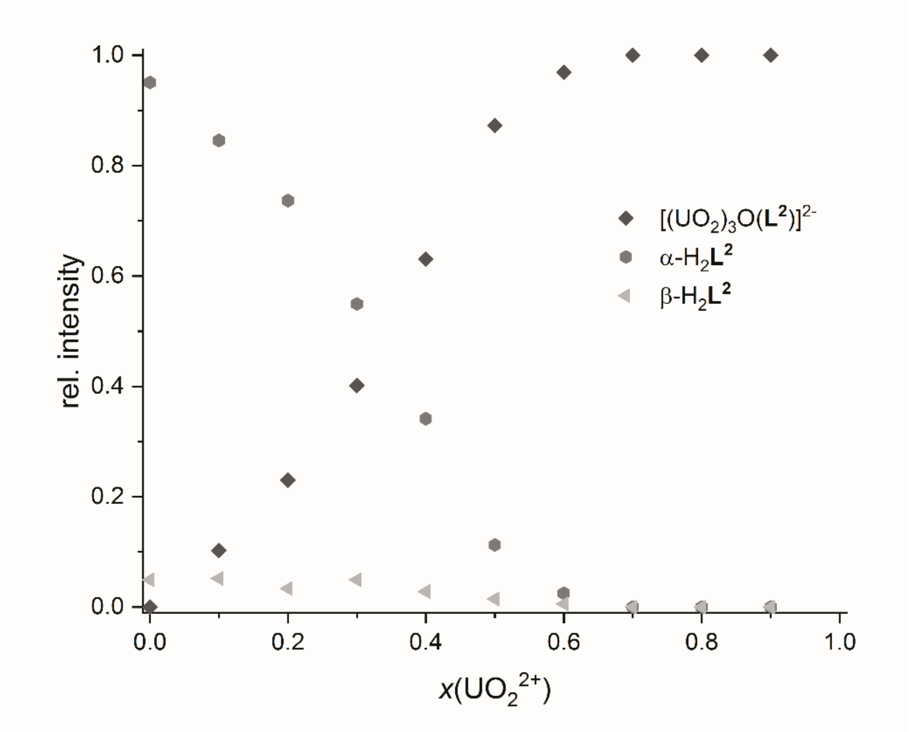

Figure S17: Relative intensities of the C17-H resonance for the species shown with respect to the increasing molar ratio ($x(UO_2^{2+})$).

S3.2 Lewis acid induced hydrolysis of H_2**L**[1]

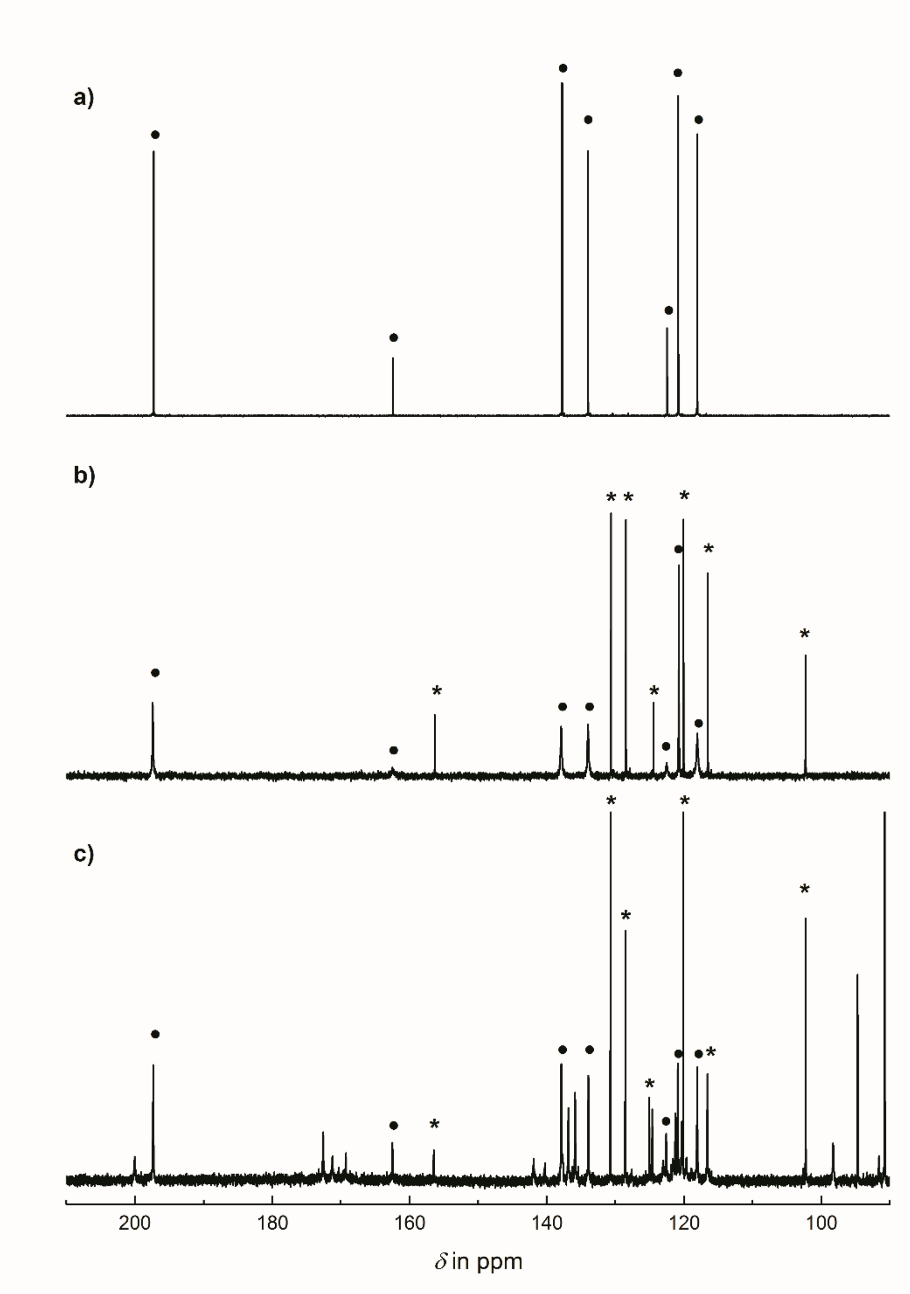

Figure S18: $^{13}C\{^{1}H\}$-NMR(125 MHz) spectra of a) Salicylic aldehyde; b) Salicylic aldehyde and $UO_2(NO_3)_2$•$6H_2O$ (M:L = 1:1); c) Reaction mixture of H_2**L**[1] and $UO_2(NO_3)_2$•$6H_2O$ in MeOD-*d4*. Resonances attributed to unbound salicylic aldehyde marked with "•", resonances attributed to metal bound salicylic aldehyde marked with "*".

S3.3 MS-spectra

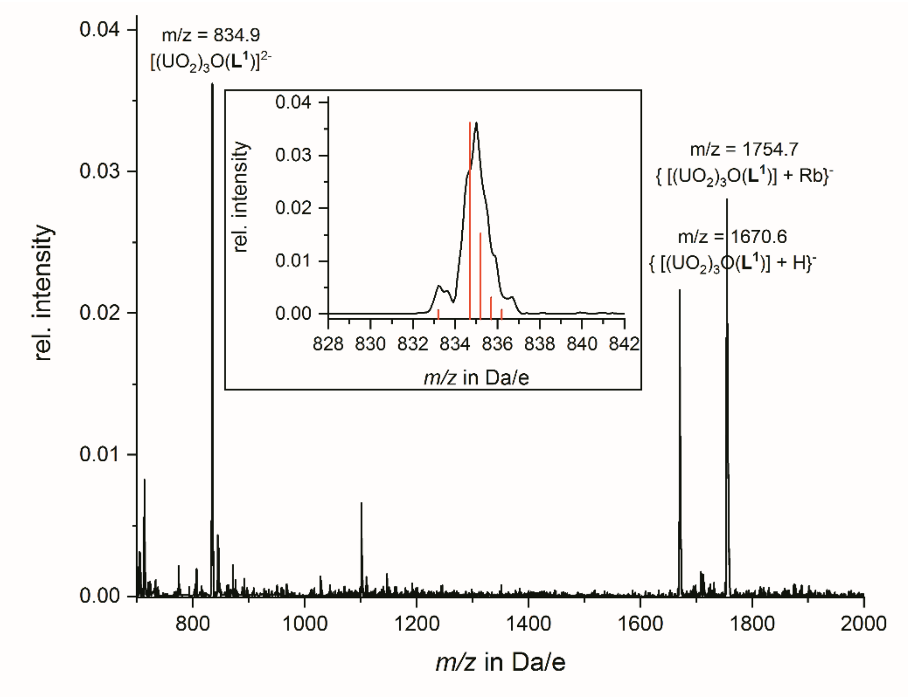

Figure S19: ESI –ve spectrum of [[Rb(18-crown-6)]₂**1**.

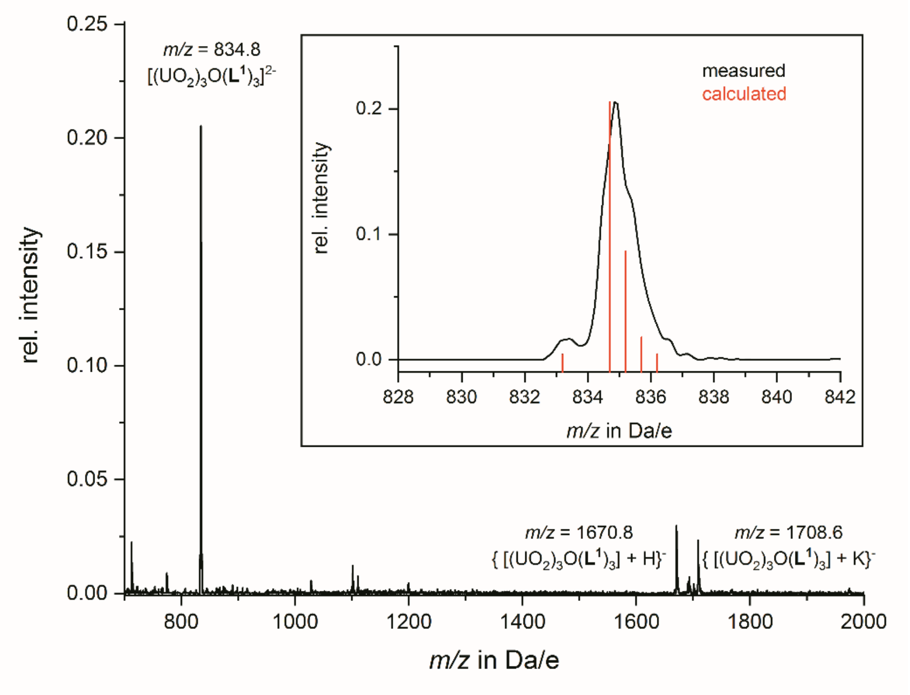

Figure S20: ESI –ve spectrum of [K(18-crown-6)]₂**1**.

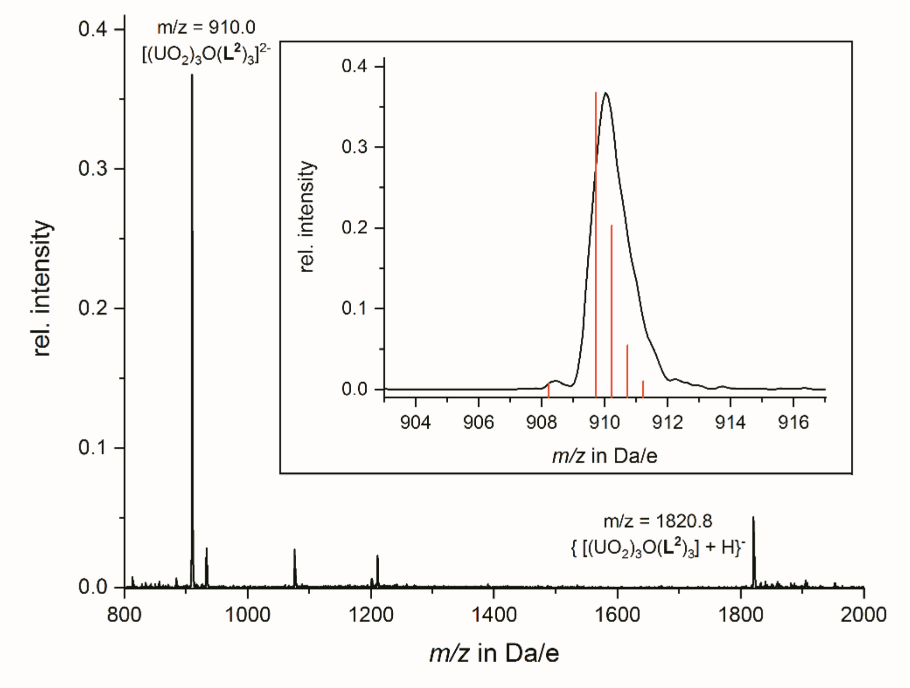

Figure S21: ESI –ve spectrum of [[Cs(18-crown-6)]₂**2**.

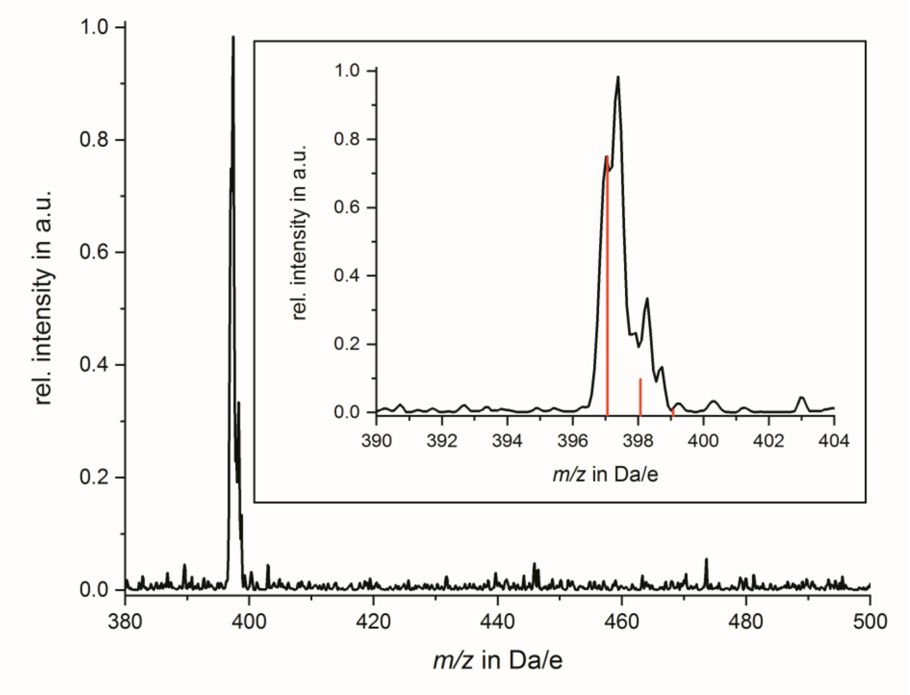

Figure S22: ESI +ve spectrum of [[Cs(18-crown-6)]₂**1**.

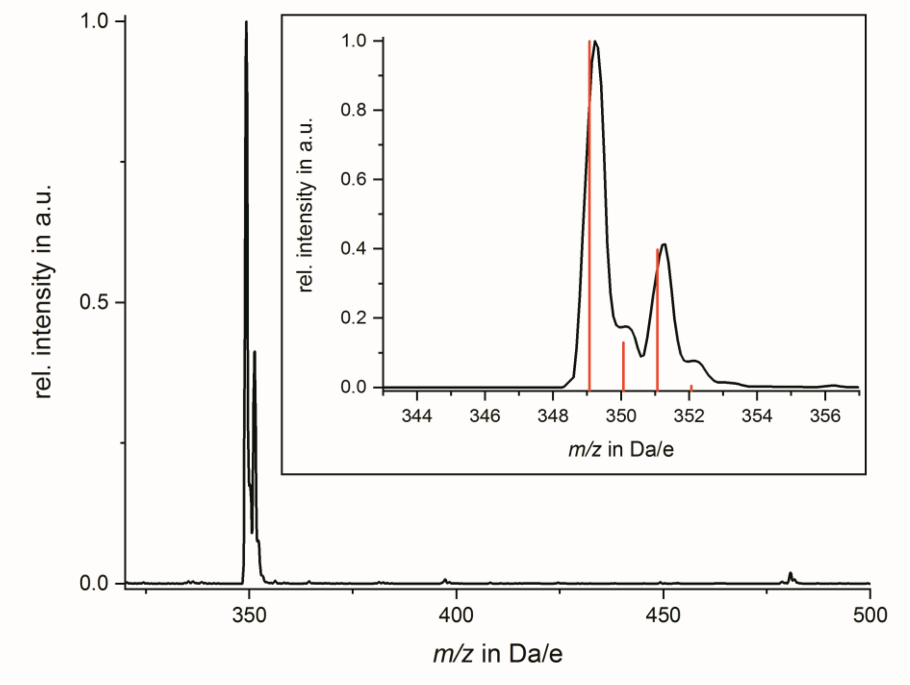

Figure S23: ESI +ve spectrum of [[Rb(18-crown-6)]$_2$**1**.

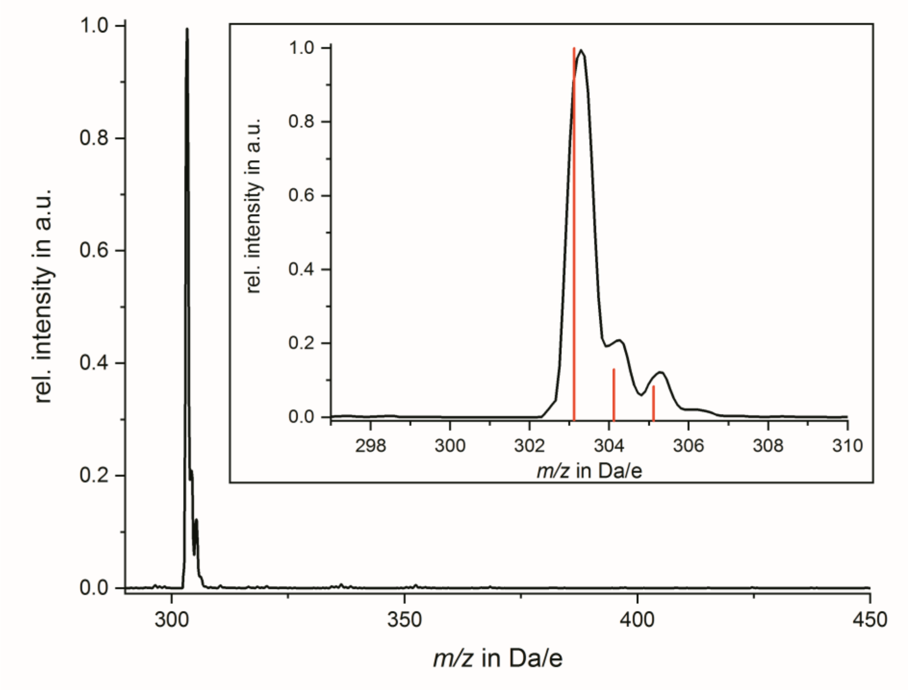

Figure S24: ESI +ve spectrum of [[K(18-crown-6)]$_2$**1**.

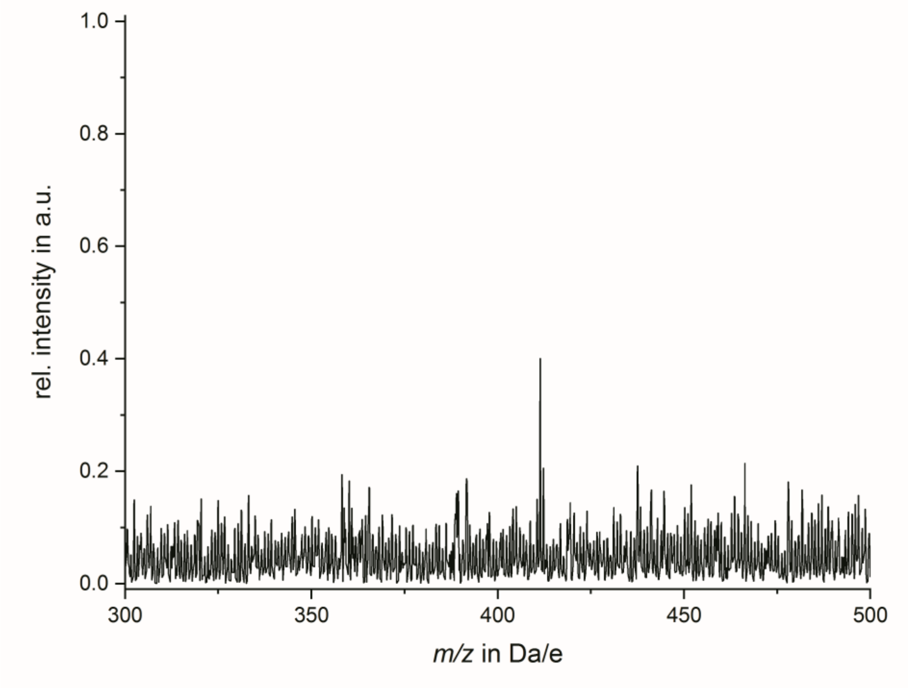

Figure S25: ESI –ve spectrum of [[Cs(18-crown-6)]$_2$**2**.

S4. Crystallographic Details

S4.1 Crystallographic Refinement

Suitable single crystals were coated with Paratone-N oil or Fomblin Y25 PFPE oil, mounted using a glass fiber and frozen in the cold nitrogen stream. X-ray diffraction data were collected 100 K on a Rigaku Oxford Diffraction SuperNova diffractometer using Cu Kα radiation (λ = 1.54184 Å) generated by a micro-focus source. The data reduction and absorption correction were performed using CrysAlisPro,[7]. For further crystal and data collection details see Table S1. The structures were solved using Olex2[8] with the SHELXT package[9] and were refined with SHELXS[10]. Images of the structures were produced with Olex2[8] software.
All structures of the Uranium complexes exhibit large electron density holes and peaks close to the heavy metal atoms as artefacts. Although absorption correction has been carefully applied based on a multifaceted crystal model, those artefacts are caused by the very high absorption coefficients. The solvent molecules in the structures of 2([Cs(18-crown-6)]$_2$**1**)•9DMF•4H_2O, [Rb(18-crown-6)]$_2$**1**•3DMF•2H_2O, [Cs(18-crown-6)]$_2$**2**•DMF•H_2O are disordered causing diffuse electron density maxima. Therefore, they had to be restrained by ISOR, SIMU and FLAT commands or even be refined isotropically. Hydrogen atoms were added to the structure models on calculated positions using the riding model. In some case they had to be restrained by DFIX commands for an orientation towards the most reasonable acceptor.

Table S1: Crystallographic data and details of the structure refinements of compounds H_2**L**^{2}, $[Cs]_2$**1**•2MeOH, 2($[Cs(18\text{-crown-}6)]_2$**1**)•9DMF•4$H_2O$, $[Rb(18\text{-crown-}6)]_2$**1**•3DMF•2$H_2O$, $[Cs(18\text{-crown-}6)]_2$**2**•DMF•$H_2O$

	H_2**L**^{2}	$[Cs]_2$**1**·2MeOH	2($[Cs(18\text{-crown-}6)]_2$**1**•9DMF•4$H_2O$
**Empirical formula**	$C_{17}H_{19}NO_6$	$C_{41}H_{53}Cs_2N_3O_{27}U_3$	$C_{153}H_{257}Cs_4N_{15}O_{87}U_6$
**FW (g mol⁻¹)**	333.33	1999.77	5658.54
**Temperature (K)**	100.1(7)	100.0(6)	100(1)
**Crystal system**	triclinic	orthorhombic	triclinic
**Space group**	*P*1	$P2_12_12_1$	*P*1
a **(Å)**	4.86457(10)	14.49368(9)	15.80763(14)
b **(Å)**	6.59200(13)	16.08227(12)	16.7259(2)
c **(Å)**	12.1222(3)	22.60717(16)	21.4309(2)
α **(°)**	97.7233(17)	90	79.0266(9)
β **(°)**	99.5183(18)	90	85.7287(8)
γ **(°)**	98.9733(17)	90	63.1642(11)
V **(Å³)**	373.528(14)	5269.53(6)	4963.17(10)
**Z**	1	4	1
**ρ_{calc} (g cm⁻³)**	1.482	2.521	1.893
**μ (mm⁻¹)**	0.947	37.077	20.047
**F(000)**	176.0	3688.0	2748.0
**Crystal size (mm⁻³)**	0.242 x 0.059 x 0.026	0.145 x 0.043 x 0.043	0.198 x 0.08 x 0.043
**Radiation**	$CuK_α$ (λ = 1.54184)	$CuK_α$ (λ = 1.54184)	$CuK_α$ (λ = 1.54184)
**2Θ range for data collection (°)**	7.496 to 153.428	6.746 to 153.672	4.2 to 153.636
**Index ranges**	-6 ≤ h ≤ 6, -8 ≤ k ≤ 8, -15 ≤ l ≤ 15	-18 ≤ h ≤ 11, -20 ≤ k ≤ 19, -28 ≤ l ≤ 28	-19 ≤ h ≤ 19, -21 ≤ k ≤ 19, -26 ≤ l ≤ 26
**Reflections collected**	5748	48389	124368
**Independent reflections**	2799 [R_{int} = 0.0150, R_{sigma} = 0.0199]	11033 [R_{int} = 0.0371, R_{sigma} = 0.0229]	39322 [R_{int} = 0.0399, R_{sigma} = 0.0390]
**Data/restraints/ parameters**	2799/3/274	11033/5/700	39322/565/2364
**Goodness-of-fit on F²**	1.069	1.054	1.041
**Final R indexes [I ≥ 2σ (I)]**	R_1 = 0.0248, wR_2 = 0.0673	R_1 = 0.0283, wR_2 = 0.0740	R_1 = 0.0318, wR_2 = 0.0823
**Final R indexes [all data]**	R_1 = 0.0249, wR_2 = 0.0677	R_1 = 0.0284, wR_2 = 0.0741	R_1 = 0.0325, wR_2 = 0.0828
**Largest diff. peak/hole (*e* Å⁻³)**	0.20/-0.19	1.36/-2.78	1.48/-1.26
**Flack parameter**	0.04(6)	-0.017(5)	-0.018(4)
**CCDC**	2055872	2055873	2055875

	$[Rb(18\text{-crown-}6)]_2$**1**•3DMF•2$H_2O$	$[Cs(18\text{-crown-}6)]_2$**2**•DMF•$H_2O$
**Empirical formula**	$C_{72}H_{118}N_6O_{42}Rb_2U_3$	$C_{87}H_{129}Cs_2N_7O_{42}U_3$
**FW (g mol⁻¹)**	2624.75	2924.87
**Temperature (K)**	102(2)	100.1(8)
**Crystal system**	triclinic	monoclinic
**Space group**	*P*1	$P2_1$
a **(Å)**	15.4702(2)	17.5480(3)
b **(Å)**	16.7672(3)	15.45477(14)
c **(Å)**	21.2182(2)	20.6768(3)
α **(°)**	95.3141(12)	90
β **(°)**	95.7532(11)	93.9593(15)
γ **(°)**	117.2420(16)	90
V **(Å³)**	4808.66(14)	5594.19(14)
**Z**	2	2
**ρ_{calc} (g cm⁻³)**	1.813	1.736
**μ (mm⁻¹)**	16.053	17.800
**F(000)**	2556.0	2844.0
**Crystal size (mm⁻³)**	0.14 x 0.059 x 0.033	0.166 x 0.127 x 0.072
**Radiation**	$CuK_α$ (λ = 1.54184)	$CuK_α$ (λ = 1.54184)
**2Θ range for data collection (°)**	6.002 to 153.62	4.284 to 153.772
**Index ranges**	-19 ≤ h ≤ 19, -21 ≤ k ≤ 21, -26 ≤ l ≤ 26	-21 ≤ h ≤ 22, -12 ≤ k ≤ 19, -25 ≤ l ≤ 25
**Reflections collected**	95832	62896
**Independent reflections**	36046 [R_{int} = 0.0498, R_{sigma} = 0.0539]	18184 [R_{int} = 0.0444, R_{sigma} = 0.0358]
**Data/restraints/ parameters**	36046/340/2273	18184/895/1273
**Goodness-of-fit on F²**	1.063	1.120
**Final R indexes [I ≥ 2σ (I)]**	R_1 = 0.0697, wR_2 = 0.1825	R_1 = 0.0721, wR_2 = 0.1859
**Final R indexes [all data]**	R_1 = 0.0725, wR_2 = 0.1860	R_1 = 0.0733, wR_2 = 0.1866
**Largest diff. peak/hole (*e* Å⁻³)**	0.20/-0.19	1.36/-2.78
**Flack parameter**	0.04(6)	-0.017(5)
**CCDC**	2055874	2055871

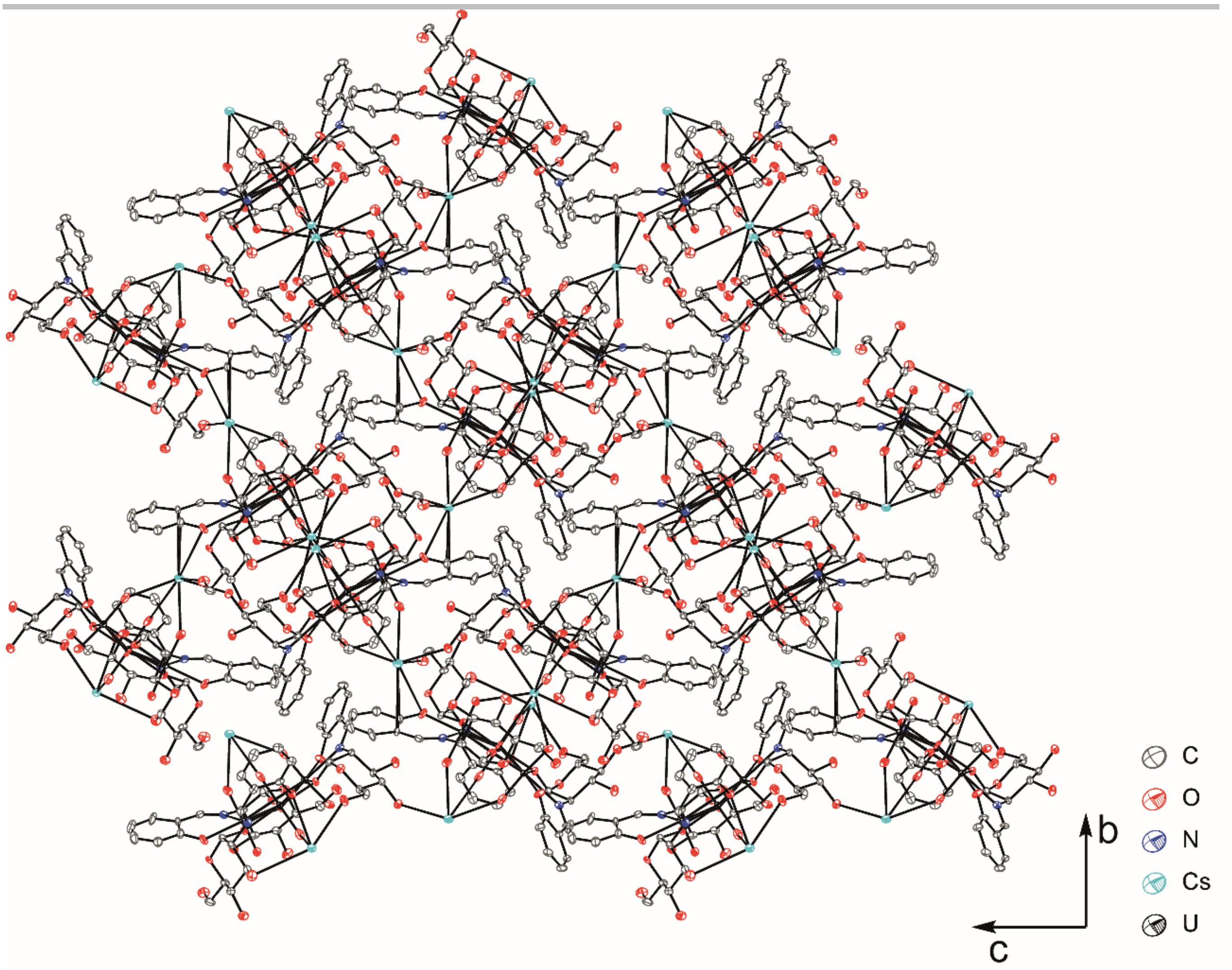

Figure S26: Three dimensional network of $[Cs]_2$**1**•2MeOH, projected in a.

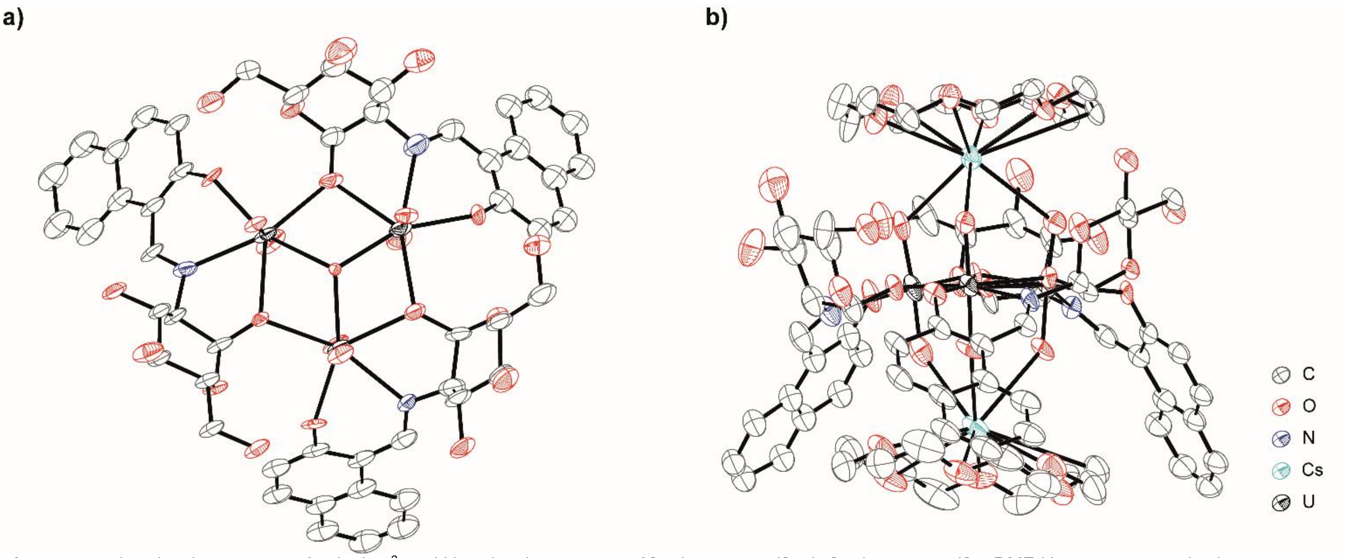

Figure S27: a) Molecular structure of anionic **2**$^{2-}$ and b) molecular structure of $[Cs(18\text{-crown-}6)]_2$**2** in $[Cs(18\text{-crown-}6)]_2$**2**•DMF•$H_2O$, H atoms and solvate molecules are omitted for clarity.

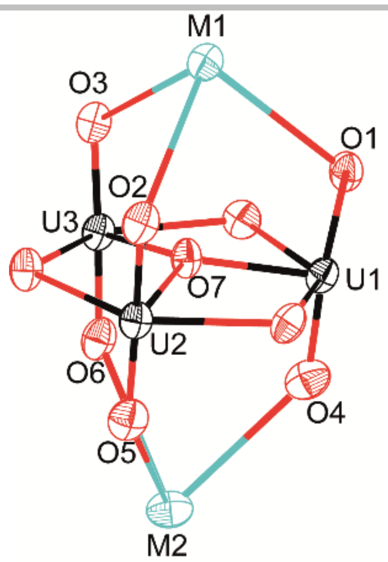

Figure S28: Central uranyl unit in 2([Cs(18-crown-6)]$_2$**1**)•9DMF•4H_2O, [Rb(18-crown-6)]$_2$**1**•3DMF•2H_2O, [Cs(18-crown-6)]$_2$**2**•DMF•H_2O , atoms labelled accordingly.

Table S2: Selected angles and bond lengths of [Cs]$_2$**1**•2MeOH, 2([Cs(18-crown-6)]$_2$**1**)•9DMF•4H_2O, [Rb(18-crown-6)]$_2$**1**•3DMF•2H_2O, [Cs(18-crown-6)]$_2$**2**•DMF•H_2O

	[Cs]$_2$**1**·2MeOH	2([Cs(18-crown-6)]$_2$**1**•9DMF•4H_2O	[Rb(18-crown-6)]$_2$**1**•3DMF•2H_2O	[Cs(18-crown-6)]$_2$**2**•DMF•H_2O
(O1-U1-O4)	173.2(4)	176.0(4)	177.4(10)	174.7(3)
(O2-U2-O5)	174.5(3)	175.9(4)	175.9(8)	175.7(4)
(O3-U3-O6)	176.7(3)	175.0(4)	178.7(9)	176.7(4)
U1-O1	1.828(8)	1.804(8)	1.814(15)	1.817(18)
U1-O4	1.799(9)	1.783(9)	1.75(2)	1.824(16)
U2-O2	1.834(8)	1.811(8)	1.814(16)	1.811(15)
U2-O5	1.795(9)	1.787(9)	1.754(19)	1.806(16)
U3-O3	1.797(8)	1.810(8)	1.838(17)	1.805(19)
U3-O6	1.787(9)	1.789(9)	1.762(18)	1.81(2)
O1-O2	4.526(10)	4.357	4.291	4.439
O1-O3	4.545(11)	4.292	4.215	4.375
O2-O3	4.475(11)	4.317	4.223	4.303
O4-O5	3.426(11)	3.548	3.515	3.462
O4-O6	3.347(11)	3.623	3.568	3.511
O5-O6	3.439(11)	3.590	3.523	3.667
M1-O1	3.192(8)	3.155(9)	3.087(18)	3.210(18)
M1-O2	7.426(8)	3.198(7)	3.093(16)	3.140(16)
M1-O3	7.476(8)	3.063(7)	2.928(17)	3.096(16)
M1-O7	6.099(7)	3.383(7)	3.285(12)	3.315(7)
M2-O4	3.544(9)	3.007(9)	2.92(2)	3.056(15)
M2-O5	3.026(9)	3.050(8)	3.00(4)	2.965(17)
M2-O6	2.966(8)	3.059(10)	2.955(19)	3.03(2)
M2-O7	4.640(8)	4.310(7)	4.205	4.386(7)

References

[1] J. C. Irvine, J. C. Earl, *J. Chem. Soc., Trans.*,**1922**, *121*, 2376-2381.

[2] Z. E. Jolles, W. T. J. Morgan, *Biochem. J*, **1940**, *34*, 1183-1190.

[3] P. S. Clough, D. Dollimore, P. Grundy, *J. inorg. Nucl. Chem.*, **1969**, *31*, 361-370.

[4] A. Lossin, G. Meyer, *Z. anorg. allg. Chem.*, **1993**, *619*, 1462-14647.

[5] R. F. Culmo, *The Elemental Analysis of Various Classes of Chemical Compounds Using CHN*, PerkinElmer, **2015**.

[6] V. P. Fadeeva, V. D. Tikhova, O. N. Nikulicheva, *J. Anal. Chem.*, **2008**, *63*, 1094-1106.

[7] CrysAlisPRO, Oxford Diffraction/Agilent Technologies UK Ltd, Yarnton, England.

[8] O. V. Dolomanov, L. J. Bourhis, R. J. Gildea, J. A. K. Howard and H. Puschmann, *J. Appl. Crystallogr.*, **2009**, *42*, 339.

[9] G. M. Sheldrick, *Acta Crystallogr. Sect. A Found. Adv.*, **2015**, *71*, 3–8.

[10] G. M. Sheldrick, *Acta Crystallogr. Sect. C Struct. Chem.* **2015**, *71*, 3–8.
